# Survival improvement over time in renal cell carcinoma treated with nephrectomy: A longitudinal propensity score‐matched study

**DOI:** 10.1111/iju.15610

**Published:** 2024-10-28

**Authors:** Kenjiro Kishitani, Satoru Taguchi, Koji Tanaka, Tetsuya Danno, Takahiro Oshina, Yoichi Fujii, Jun Kamei, Yoshiyuki Akiyama, Shigenori Kakutani, Yusuke Sato, Yuta Yamada, Aya Niimi, Daisuke Yamada, Haruki Kume

**Affiliations:** ^1^ Department of Urology, Graduate School of Medicine The University of Tokyo Bunkyo‐ku Tokyo Japan

**Keywords:** laparoscopic, nephrectomy, propensity score matching, renal cell carcinoma, robotic

## Abstract

**Objective:**

Surgical treatment for renal cell carcinoma (RCC) has drastically evolved for the past 30 years. However, survival outcomes of RCC according to times have not been fully elucidated, especially in the real‐world setting. This study aimed to assess the survival improvement over time in RCC treated with nephrectomy by analyzing a longitudinal cohort using propensity score matching (PSM).

**Methods:**

We retrospectively reviewed 960 patients with RCC who underwent radical or partial nephrectomy between 1981 and 2018. Patients were divided into two groups according to the time of surgery (1981–1999 vs. 2000–2018). Using PSM, overall survival (OS), cancer‐specific survival (CSS), and recurrence‐free survival (RFS) were compared between the two groups.

**Results:**

Overall, 255 and 705 patients underwent surgery in the earlier (1981–1999) and recent (2000–2018) eras, and PSM derived a matched cohort of 466 patients (233 patients per each group). All patients in the earlier era cohort received open surgeries, whereas about a half (47.4%) of patients in the recent era cohort received minimally‐invasive (laparoscopic/robotic) surgeries. After PSM, 137 (29.4%) patients developed recurrence, 105 (22.5%) died of RCC, and 113 (24.2%) died from other causes, with a median follow‐up period of 90 months. The recent era cohort had significantly longer OS, CSS, and RFS than the earlier era cohort.

**Conclusions:**

Patients with RCC treated in the recent era (2000–2018) showed significantly longer survival than those treated in the earlier era (1981–1999). The improved survival might be attributable to the prevalence of minimally‐invasive (laparoscopic/robotic) surgeries.

**Abbreviations & Acronyms**
CSS: cancer‐specific survival
IQR: interquartile range
OS: overall survival
PSM: propensity score matching
RCC: renal cell carcinoma
RFS: recurrence‐free survival

## INTRODUCTION

In 2022, 434 840 patients worldwide were diagnosed with renal cell carcinoma (RCC), resulting in more than 155 953 deaths.^1^ While the main therapeutic approach for early stage RCC is surgical resection, surgical techniques for RCC have drastically evolved for the past 30 years. Since the 1860s, open radical or partial nephrectomy has been performed for more than 100 years.^2^, ^3^ However, in the 1990s, laparoscopic radical^4^ or partial^5^ nephrectomy was introduced as a less invasive procedure. Furthermore, in the 2000s, robot‐assisted laparoscopic partial nephrectomy was launched, which is currently the gold standard treatment for early stage RCC.^6^ Nevertheless, survival outcomes of RCC according to times have not been fully elucidated, especially in the real‐world setting.^7^ Therefore, the present study aimed to assess the survival improvement over time in RCC treated with nephrectomy by analyzing a longitudinal cohort using propensity score matching (PSM).

## METHODS

### Patients

This study was approved by the Institutional Review Board of the Graduate School of Medicine and Faculty of Medicine, The University of Tokyo (approval number: 3124). Because of the retrospective design of this study, the need for written informed consent was waived.

**FIGURE 1:**
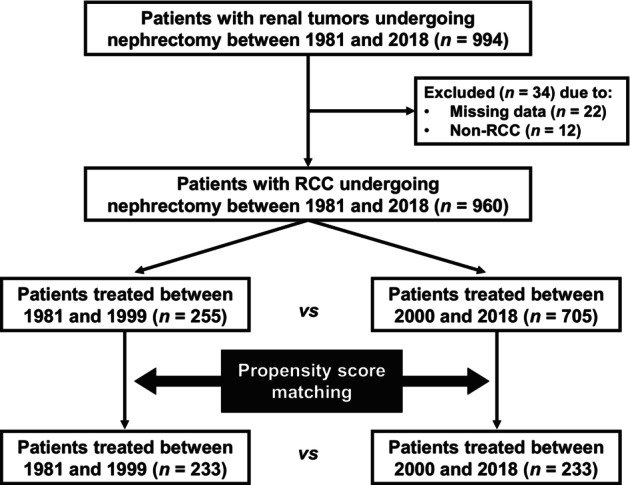
Flowchart presenting the study selection process.

We retrospectively reviewed 994 patients with renal tumors who underwent radical or partial nephrectomy at The University of Tokyo Hospital between 1981 and 2018. If a patient underwent multiple nephrectomies, the first nephrectomy was counted. We excluded 34 patients because of missing data (*n* = 22) or non‐RCC in the pathological diagnosis (*n* = 12). Eventually, 960 patients with RCC undergoing nephrectomy were identified for analysis. The cohort included 436 patients who were analyzed in our previous work.^8^ The patients were divided into two groups according to the time of surgery (1981–1999 [*n* = 255] vs. 2000–2018 [*n* = 705]). The dichotomization was based on the equal division of the study period (1981–2018, 38 years) into the first (1981–1999, 19 years), and second (2000–2018, 19 years) halves. Then PSM derived a matched cohort of 466 patients (233 patients per each group). Figure 1 illustrates a flowchart presenting the study selection process.

Pathological stage (pStage) was re‐evaluated according to the eighth TNM classification^9^ based on pathology reports, medical charts, radiogram interpretation reports, and so on.

### Endpoints and statistical analyses

We assessed the associations of clinicopathological variables, including age, sex, the time of surgery (or “era”), surgical procedure (open, laparoscopic, or robotic), nephrectomy type (radical or partial), pStage (I–IV), and histological type (clear cell or non‐clear cell), with overall survival (OS), cancer‐specific survival (CSS), and recurrence‐free survival (RFS). OS was defined as the time from surgery to death from any cause. CSS was defined as the time from surgery to death from RCC. RFS was defined as the time from surgery to recurrence or death, whichever occurred first. Median follow‐up was calculated as the median time from surgery to the last follow‐up date (either death or the last visit). Follow‐up information was obtained as of December 2023.

For PSM, multivariable logistic regression analysis was used to calculate propensity scores, and matching was conducted on the logit of the propensity score using nearest neighbor matching with a caliper of 0.20. Before and after PSM, the significance of the differences of clinicopathological variables between the two groups were evaluated using Student's *t*‐test for continuous variables and the Chi‐squared test for categorical variables. Before and after PSM, OS, CSS, and RFS were estimated using the Kaplan–Meier method and compared using the log‐rank test. Univariable and multivariable Cox proportional hazard regression analyses for OSS, CSS, and RFS were conducted before PSM. Univariable Cox proportional hazard regression analyses were also conducted after PSM. All statistical analyses were performed using JMP Pro version 17.0.0 (SAS Institute, Cary, NC, USA). *p* < 0.05 was considered significant.

## RESULTS

### Analyses of crude data before PSM

**FIGURE 2:**
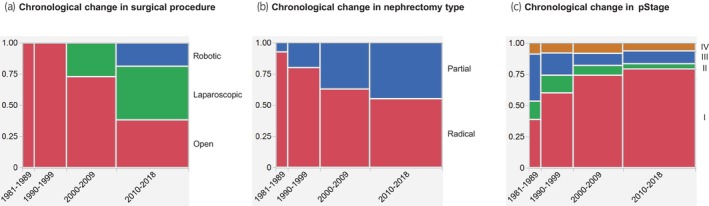
Chronological changes in (a) surgical procedure, (b) nephrectomy type, and (c) pStage before PSM (*n* = 960). PSM, propensity score matching.

The left half of Table 1 shows the characteristics of all patients (*n* = 960) before PSM. The recent era (2000–2018) cohort was significantly older, received significantly more minimally‐invasive (laparoscopic/robotic) surgeries and partial nephrectomies, and included significantly more pStage I diseases and non‐clear cell histologies than the earlier era (1981–1999) cohort. All patients in the earlier era cohort received open surgeries, whereas about a half (47.4%) of patients in the recent era cohort received minimally‐invasive (laparoscopic/robotic) surgeries. Figure 2 shows chronological changes in surgical procedure, nephrectomy type, and pStage before PSM. Minimally‐invasive (laparoscopic/robotic) surgeries, partial nephrectomies, and pStage I diseases increased with the times.

**TABLE 1:** Patients' baseline characteristics before and after PSM.

Parameter	Before PSM				After PSM			
	Total (*n* = 960)	1981–1999 (*n* = 255)	2000–2018 (*n* = 705)	*P*‐value	Total (*n* = 466)	1981–1999 (*n* = 233)	2000–2018 (*n* = 233)	*p*‐value
Age, years, median (IQR)	61 (52–69)	56 (47–64)	62 (54–71)	<0.001^a^ ^,^ *	58 (48–65)	58 (49–65)	57 (48–65)	0.920^a^
Sex, no. (%)				0.052^b^				0.736^b^
Male	713 (74.3)	201 (78.8)	512 (72.6)		365 (78.3)	181 (77.7)	184 (79.0)	
Female	247 (25.7)	54 (21.2)	193 (27.4)		101 (21.7)	52 (22.3)	49 (21.0)	
Surgical procedure, no. (%)				<0.001^b^ ^,^ *				<0.001^b^ ^,^ ^a^ ^,^ *
Open	626 (65.2)	255 (100)	371 (52.6)		353 (75.8)	233 (100)	120 (51.5)	
Laparoscopic	256 (26.7)	0 (0)	256 (36.3)		103 (22.1)	0 (0)	103 (44.2)	
Robotic	78 (8.1)	0 (0)	78 (11.1)		10 (2.1)	0 (0)	10 (4.3)	
Nephrectomy type, no. (%)				<0.001^b^ ^,^ ^a^ ^,^ *				0.537^b^
Radical	625 (65.1)	213 (83.5)	412 (58.4)		387 (83.0)	191 (82.0)	196 (84.1)	
Partial	335 (34.9)	42 (16.5)	293 (41.6)		79 (17.0)	42 (18.0)	37 (15.9)	
pStage, no. (%)				<0.001^b^ ^,^ ^a^ ^,^ *				0.692^b^
I	684 (71.3)	139 (54.5)	545 (77.3)		285 (61.2)	138 (59.2)	147 (63.1)	
II	77 (8.0)	36 (14.1)	41 (5.8)		52 (11.2)	29 (12.4)	23 (9.9)	
III	130 (13.5)	60 (23.5)	70 (9.9)		95 (20.4)	47 (20.2)	48 (20.6)	
IV	69 (7.2)	20 (7.8)	49 (7.0)		34 (7.3)	19 (8.2)	15 (6.4)	
Histological type, no. (%)				0.038^b^ ^,^ *				0.662^b^
Clear cell RCC	825 (85.9)	229 (89.8)	596 (84.5)		413 (88.6)	208 (89.3)	205 (88.0)	
Non‐clear cell RCC	135 (14.1)	26 (10.2)	109 (15.5)		53 (11.4)	25 (10.7)	28 (12.0)	

Abbreviations: IQR, interquartile range; PSM, propensity score matching; RCC, renal cell carcinoma.

^a^ Student's *t*‐test.

^b^ Chi‐squared test.

* Statistically significant.

**FIGURE 3:**
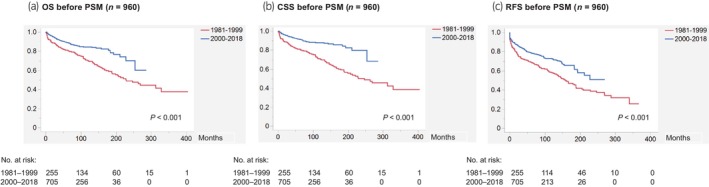
Kaplan–Meier curves depicting (a) OS, (b) CSS, and (c) RFS according to the era (1981–1999 vs. 2000–2018) before PSM (*n* = 960). CSS, cancer‐specific survival; OS, overall survival; PSM, propensity score matching; RFS, recurrence‐free survival.

In total, 241 (25.1%) patients developed recurrence, 168 (17.5%) died of RCC, and 193 (20.1%) died from other causes, with a median follow‐up period of 81 months. Figure 3 presents Kaplan–Meier curves depicting OS, CSS, and RFS according to the era (1981–1999 vs. 2000–2018) before PSM. Patients in the recent era cohort had significantly longer OS (not reached), CSS (not reached), and RFS (not reached) than those in the earlier era cohort (OS, 229 months; CSS 247 months; and RFS, 158 months). Univariable Cox proportional hazard regression analyses associated the earlier era with significantly shorter OS, CSS, and RFS, whereas multivariable analyses identified it as an independent predictor of shorter OS and CSS but not RFS (Table S1). For reference, Kaplan–Meier curves according to other clinicopathological variables before PSM are shown in Figure S1. Kaplan–Meier curves according to the era (1981–1999 vs. 2000–2018) in each pStage (I–IV) before PSM are also shown in Figure S2.

### Analyses after PSM

Table 1 shows patients' baseline characteristics before and after PSM. For PSM, all six variables listed in Table 1 were matched except for surgical procedure, because the variable fundamentally differed between the eras. The right half of Table 1 shows the characteristics of 466 patients after PSM. Baseline characteristics between the two groups (1981–1999 vs. 2000–2018) were balanced after PSM, and differences in all variables were nonsignificant except for surgical procedure. All patients in the earlier era cohort received open surgeries, whereas about a half (48.5%) of patients in the recent era cohort received minimally‐invasive (laparoscopic/robotic) surgeries.

**FIGURE 4:**
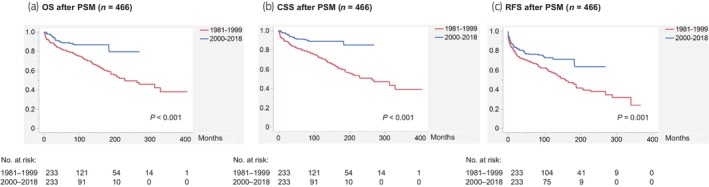
Kaplan–Meier curves depicting (a) OS, (b) CSS, and (c) RFS according to the era (1981–1999 vs. 2000–2018) after PSM (*n* = 466). CSS, cancer‐specific survival; OS, overall survival; PSM, propensity score matching; RFS, recurrence‐free survival.

After PSM, 137 (29.4%) patients developed recurrence, 105 (22.5%) died of RCC, and 113 (24.2%) died from other causes, with a median follow‐up period of 90 months. Figure 4 presents Kaplan–Meier curves depicting OS, CSS, and RFS according to the era (1981–1999 vs. 2000–2018) after PSM. Again, patients in the recent era cohort had significantly longer OS (not reached), CSS (not reached), and RFS (not reached) than those in the earlier era cohort (OS, 229 months; CSS 263 months; and RFS, 158 months). Univariable Cox proportional hazard regression analyses after PSM associated the earlier era with significantly shorter OS, CSS, and RFS. As for other variables, age (≥61 vs. ≤60 years), surgical procedure (open vs. laparoscopic/robotic), nephrectomy type (radical vs. partial), pStage (III–IV vs. I–II), and histological type (non‐clear cell vs. clear cell) were all associated with significantly shorter OS, CSS, and RFS, whereas sex (male vs. female) was not (Table S2). For reference, Kaplan–Meier curves according to other clinicopathological variables after PSM are shown in Figure S3. Kaplan–Meier curves according to the era (1981–1999 vs. 2000–2018) in each pStage (I–IV) after PSM are also shown in Figure S4.

## DISCUSSION

Clinical studies on RCC have been actively conducted, focusing on genetic characteristics,^10^, ^11^ surgical procedures,^12^, ^13^ recurrence pattern,^14^ adjuvant treatment after surgery,^8^ systemic treatment in advanced disease,^15^, ^16^, ^17^ prognostic models,^18^, ^19^ chronic kidney disease after surgery,^20^ associations with hemodialysis,^7^ and so on. In terms of the initial treatment, surgical resection is the main therapeutic approach for early stage RCC, and surgical techniques for RCC have drastically evolved for the past 30 years. More specifically, the introduction of minimally‐invasive surgeries, including laparoscopic^4^, ^5^ and robotic^6^ ones, has changed the paradigm. Although survival outcomes of RCC are thought to be improved according to times due to these therapeutic advancements, studies comparing outcomes of real‐world patients with RCC between different eras are lacking.^7^ Hence, the present study compared survival outcomes between the earlier era (1981–1999) and the recent era (2000–2018) among patients with RCC treated with nephrectomy using PSM.

Our analyses of crude data before PSM showed that minimally‐invasive (laparoscopic/robotic) surgeries, partial nephrectomies, and pStage I diseases increased as time advanced (Figure 2). Given that laparoscopic^4^, ^5^ and robotic^6^ nephrectomies were introduced in the 1990s and 2000s, respectively, no patient in the earlier era (1981–1999) cohort received these minimally‐invasive surgeries. Partial nephrectomy has become more prevalent probably due to the introduction of robot‐assisted laparoscopic partial nephrectomy.^6^ The reason for the increase of pStage I diseases might be attributable to the advancement of imaging modalities such as ultrasound, computed tomography, and magnetic resonance imaging, leading to incidental detection of small renal masses.^21^

Our survival analyses before PSM showed that the earlier era (1981–1999) was associated with significantly shorter OS, CSS, and RFS (Figure 3), while multivariable analyses identified it as an independent predictor of shorter OS and CSS but not RFS (Table S1). The last result might be attributable to unadjusted confounders such as systemic treatment for RCC. Albeit no assessment in this study, systemic therapies for RCC, including tyrosine kinase inhibitors and immune checkpoint inhibitors, have drastically evolved for the past 20 years, which resulted in survival improvement of real‐world patients with advanced RCC.^15^, ^16^, ^17^ Use of systemic treatment for postoperative recurrence might contribute to the prolongation of OS and CSS (but not of RFS) in the recent era cohort. Aside from systemic therapies, advancements in the management of comorbidities such as preoperative evaluation using the American Society of Anesthesiologists physical status classification system and focal therapy or active surveillance for surgery‐ineligible patients might also favor outcomes of the recent era cohort. Furthermore, the improvement in Japanese life expectancy over time could affect the outcomes of OS (but not CSS and RFS) in this longitudinal study. Nevertheless, our survival analyses after PSM successfully demonstrated that patients in the recent era (2000–2018) cohort had significantly longer OS, CSS, and RFS than those in the earlier era (1981–1999) cohort (Figure 4). Univariable Cox proportional hazard regression analyses after PSM also confirmed the result, as well as revealed that other variables, including surgical procedure (open vs. laparoscopic/robotic) and nephrectomy type (radical vs. partial), were associated with significantly shorter OS, CSS, and RFS (Table S2). These results highlight the survival improvement over time in RCC treated with nephrectomy, which might be attributable to the prevalence of minimally‐invasive (laparoscopic/robotic) surgeries in the last 30 years.

Real‐world evidence on this topic is very scarce. Ishihara et al. analyzed a longitudinal cohort of 3037 patients with RCC treated with nephrectomy between 1979 and 2020.^7^ Since this study focused on comparisons of RCC arising in end‐stage renal disease (ESRD‐RCC, *n* = 305) with sporadic RCC (*n* = 2732), the authors assessed stage at diagnosis, surgical procedure, survival outcomes, and so on. They observed shorter CSS of ESRD‐RCC than sporadic RCC, as well as improved CSS in sporadic RCC but not in ESRD‐RCC according to time. They eventually concluded that the shorter CSS of ESRD‐RCC than sporadic RCC might be attributed to the improved outcome of sporadic RCC in recent years rather than a decline in outcomes for ESRD‐RCC.^7^ Aside from this paper, our study might be the first to assess survival improvement in RCC treated with nephrectomy for a long period of time (~40 years) using PSM. Although the present study is limited by its single‐center retrospective design, we believe that it will add additional evidence in the field.

In conclusion, patients with RCC treated in the recent era (2000–2018) showed significantly longer survival than those treated in the earlier era (1981–1999). The improved survival might be attributable to the prevalence of minimally‐invasive (laparoscopic/robotic) surgeries.

## AUTHOR CONTRIBUTIONS

**Kenjiro Kishitani:** Conceptualization; formal analysis; writing – original draft; project administration; methodology. **Satoru Taguchi:** Conceptualization; formal analysis; writing – original draft; project administration; methodology. **Koji Tanaka:** Data curation; writing – review and editing. **Tetsuya Danno:** Data curation; writing – review and editing. **Takahiro Oshina:** Data curation; writing – review and editing. **Yoichi Fujii:** Data curation; writing – review and editing. **Jun Kamei:** Data curation; writing – review and editing. **Yoshiyuki Akiyama:** Data curation; writing – review and editing. **Shigenori Kakutani:** Data curation; writing – review and editing. **Yusuke Sato:** Data curation; writing – review and editing. **Yuta Yamada:** Data curation; writing – review and editing. **Aya Niimi:** Data curation; writing – review and editing. **Daisuke Yamada:** Data curation; writing – review and editing. **Haruki Kume:** Supervision; writing – review and editing.

## CONFLICT OF INTEREST STATEMENT

Haruki Kume and Yusuke Sato are Editorial Board members of International Journal of Urology and co‐authors of this article. To minimize bias, they were excluded from all editorial decision making related to the acceptance of this article for publication. The other authors declare that they have no competing interests.

## INFORMED CONSENT

Because of the retrospective design of the study, the need for written informed consent was waived.

## REGISTRY AND THE REGISTRATION NO. OF THE STUDY/TRIAL

None.

## ANIMAL STUDIES

None.

## FUNDING INFORMATION

We received no funding/grant support for this study.

## ETHICS STATEMENT

This study was approved by the Institutional Review Board of the Graduate School of Medicine and Faculty of Medicine, The University of Tokyo (approval number: 3124).

## Supporting information


Figure S1.

Figure S2.

Figure S3.

Figure S4.

Table S1.

Table S2.

## Data Availability

The dataset analyzed in the present study is available from the corresponding author upon reasonable request.
