# Supplementary material for: Survival improvement over time in renal cell carcinoma treated with nephrectomy: A longitudinal propensity score‐matched study
Source: Int J Urol. 2024 Oct 28;32(2):145–50. doi: 10.1111/iju.15610 (PMC11803181; doi:10.1111/iju.15610)
Supplement: Supplementary file 1 — Figure S1. [file IJU-32-145-s001.pdf]

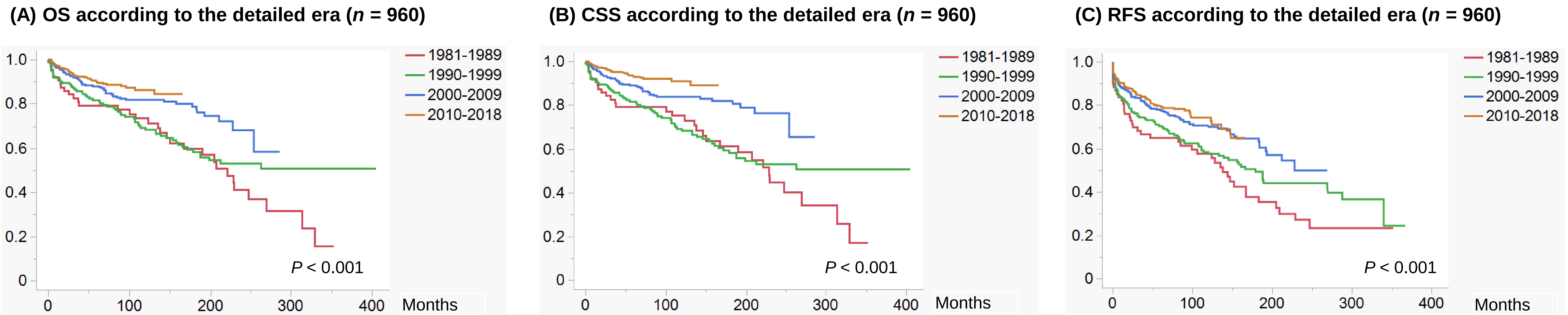


No. at risk:

| | (A) 0 | 100 | 200 | 300 | 400 | (B) 0 | 100 | 200 | 300 | 400 | (C) 0 | 100 | 200 | 300 | 400 |
|---|---|---|---|---|---|---|---|---|---|---|---|---|---|---|---|
| 1981–1989 | 69 | 41 | 19 | 4 | 0 | 69 | 41 | 19 | 4 | 0 | 69 | 33 | 14 | 2 | 0 |
| 1990–1999 | 186 | 93 | 41 | 11 | 1 | 186 | 93 | 41 | 11 | 1 | 186 | 81 | 32 | 8 | 0 |
| 2000–2009 | 287 | 153 | 36 | 0 | 0 | 287 | 153 | 36 | 0 | 0 | 287 | 130 | 26 | 0 | 0 |
| 2010–2018 | 418 | 103 | 0 | 0 | 0 | 418 | 103 | 0 | 0 | 0 | 418 | 83 | 0 | 0 | 0 |

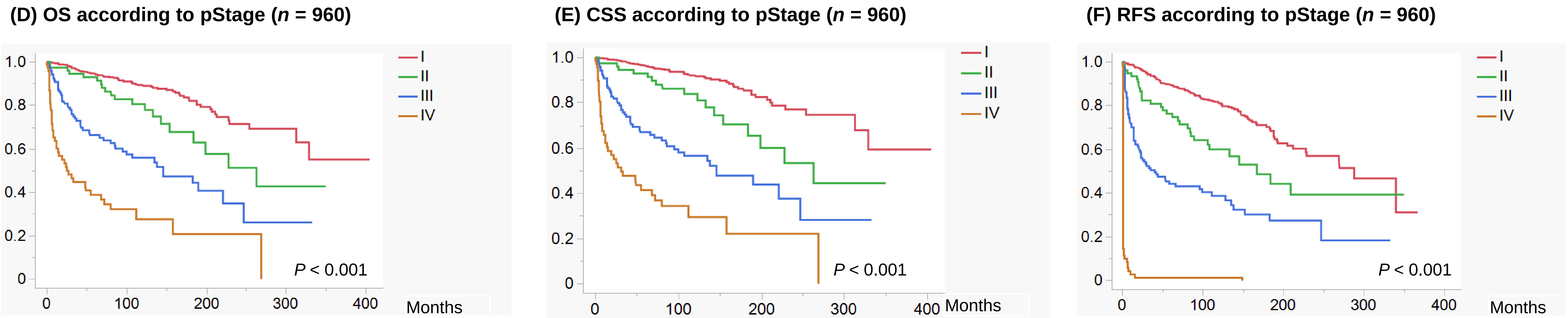


No. at risk:

| | (D) 0 | 100 | 200 | 300 | 400 | (E) 0 | 100 | 200 | 300 | 400 | (F) 0 | 100 | 200 | 300 | 400 |
|---|---|---|---|---|---|---|---|---|---|---|---|---|---|---|---|
| I | 684 | 296 | 75 | 12 | 1 | 684 | 296 | 75 | 12 | 1 | 684 | 265 | 57 | 7 | 0 |
| II | 77 | 41 | 10 | 2 | 0 | 77 | 41 | 10 | 2 | 0 | 77 | 32 | 9 | 2 | 0 |
| III | 130 | 43 | 8 | 1 | 0 | 130 | 43 | 8 | 1 | 0 | 130 | 29 | 6 | 1 | 0 |
| IV | 69 | 10 | 3 | 0 | 0 | 69 | 10 | 3 | 0 | 0 | 69 | 1 | 0 | 0 | 0 |

**(G) OS according to age ($n$ = 960)**

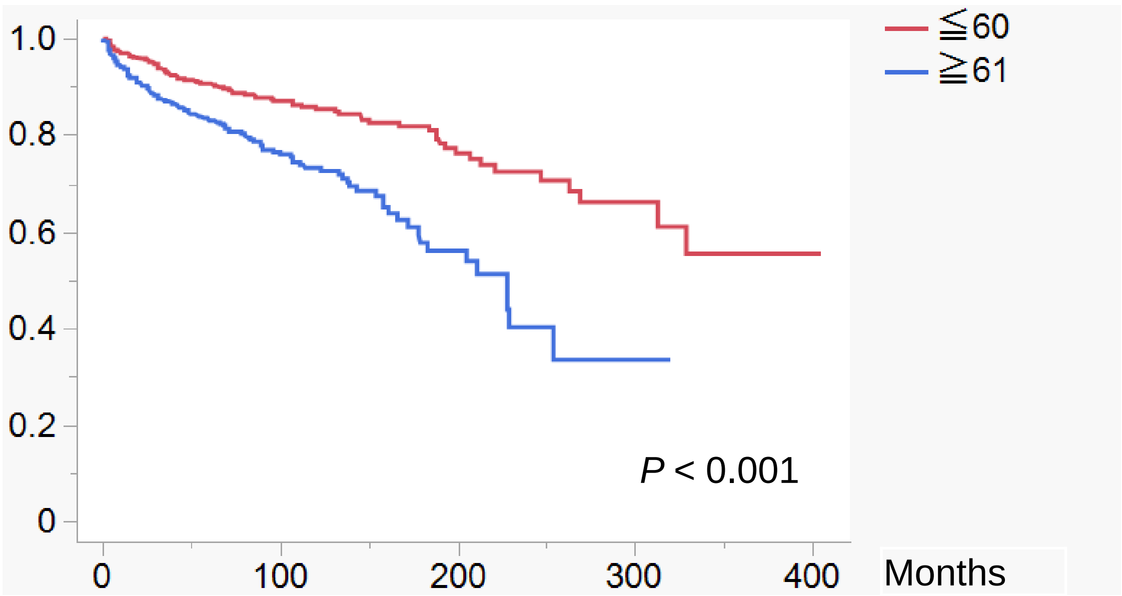


No. at risk:

| | | | | | |
|---|---|---|---|---|---|
| ≦60 | 477 | 234 | 69 | 13 | 1 |
| ≧61 | 483 | 156 | 27 | 2 | 0 |

**(H) CSS according to age ($n$ = 960)**

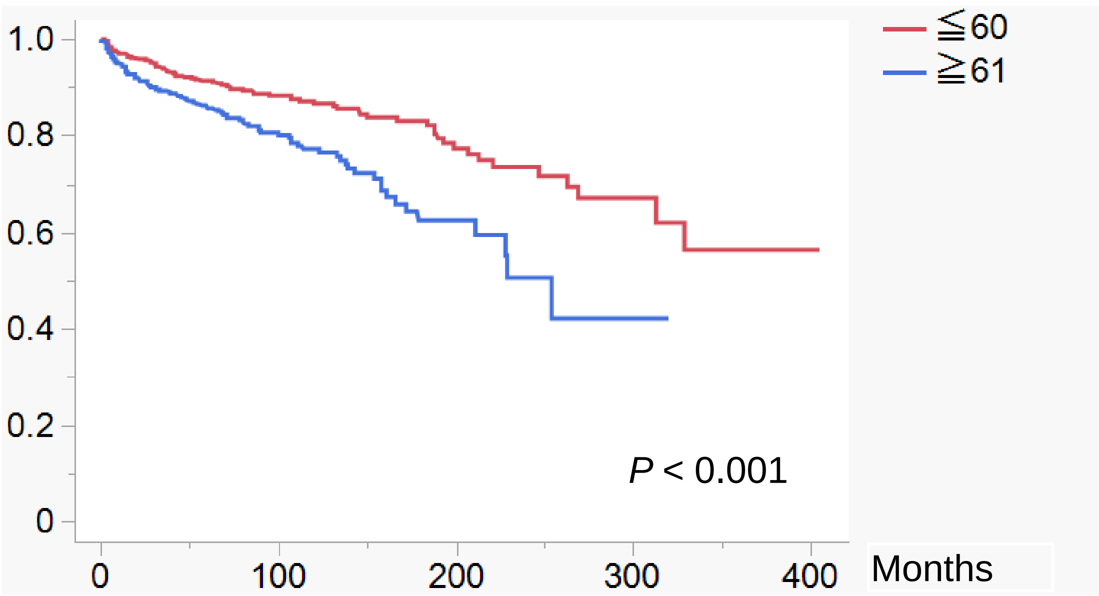


No. at risk:

| | | | | | |
|---|---|---|---|---|---|
| ≦60 | 477 | 234 | 69 | 13 | 1 |
| ≧61 | 483 | 156 | 27 | 2 | 0 |

**(I) RFS according to age ($n$ = 960)**

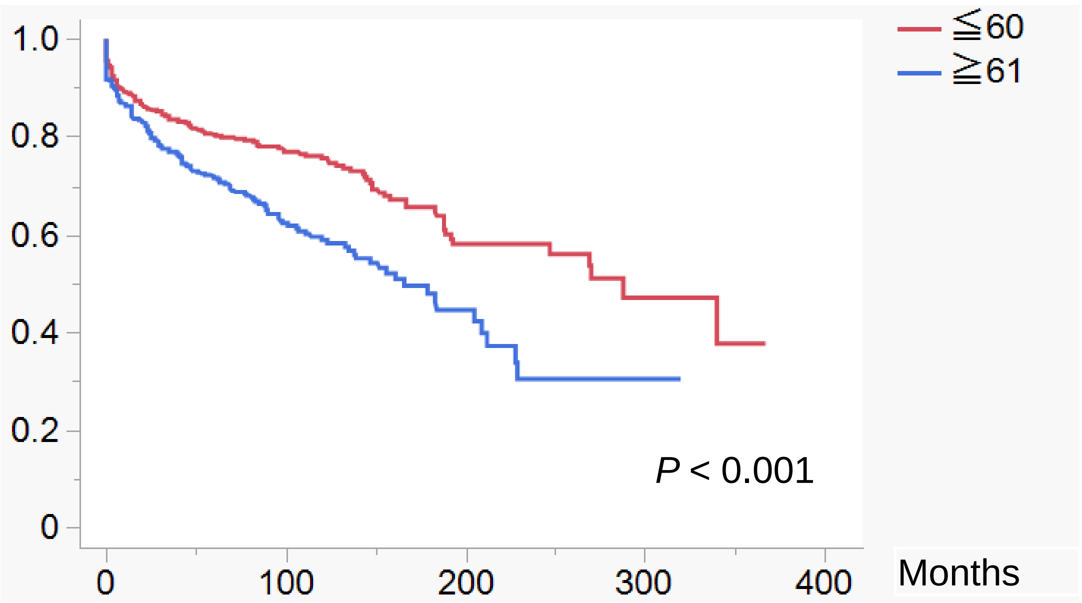


No. at risk:

| | | | | | |
|---|---|---|---|---|---|
| ≦60 | 477 | 202 | 51 | 8 | 0 |
| ≧61 | 483 | 125 | 21 | 1 | 0 |

**(J) OS according to sex ($n$ = 960)**

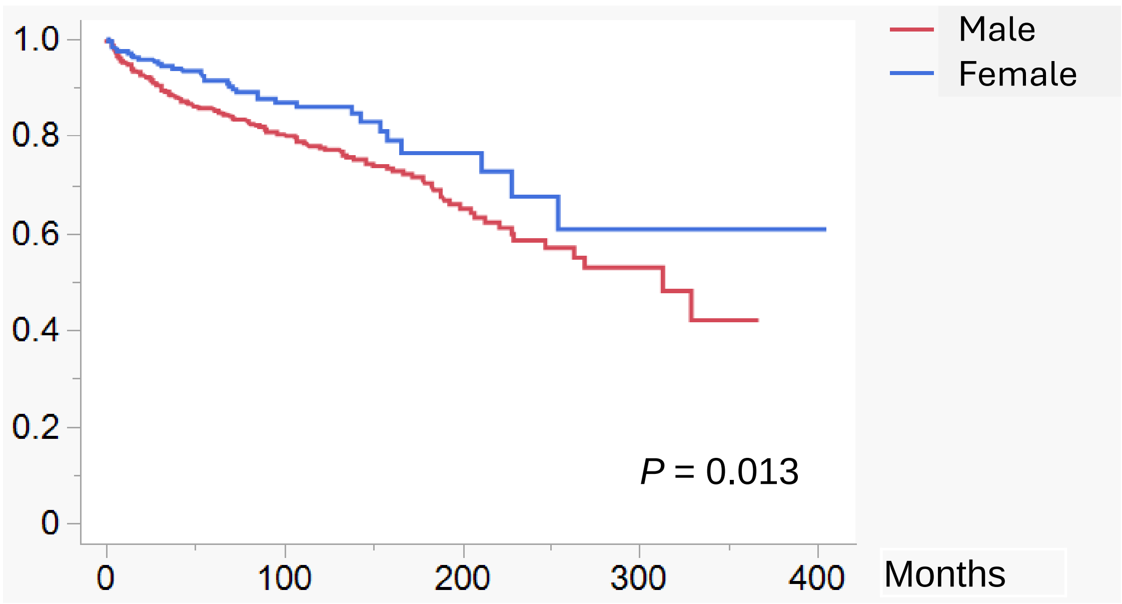


No. at risk:

| | | | | | |
|---|---|---|---|---|---|
| Male | 713 | 280 | 74 | 12 | 0 |
| Female | 247 | 110 | 22 | 3 | 1 |

**(K) CSS according to sex ($n$ = 960)**

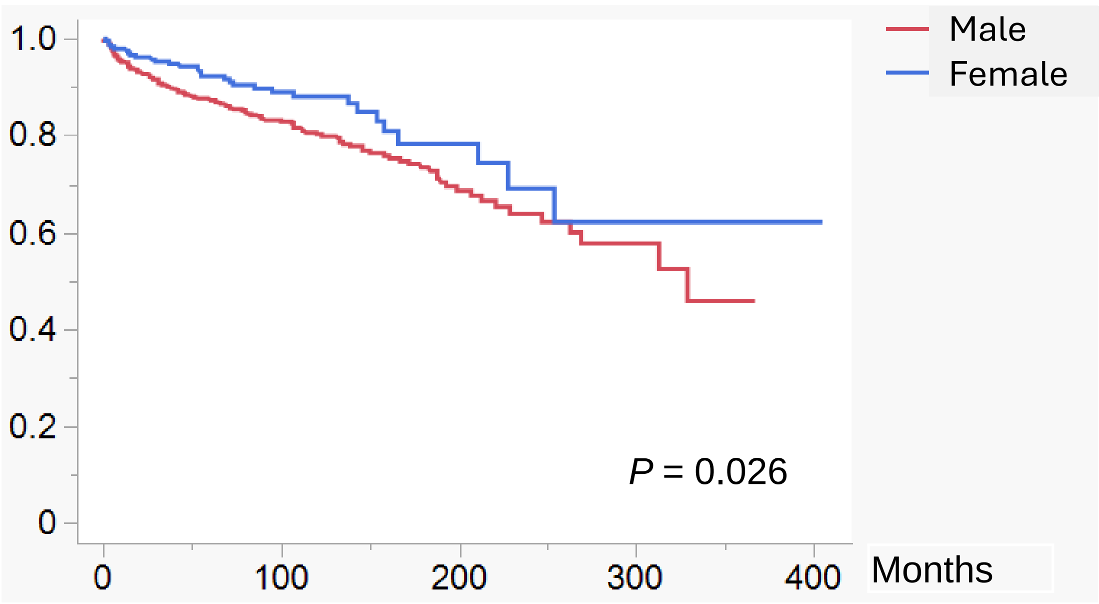


No. at risk:

| | | | | | |
|---|---|---|---|---|---|
| Male | 713 | 280 | 74 | 12 | 0 |
| Female | 247 | 110 | 22 | 3 | 1 |

**(L) RFS according to sex ($n$ = 960)**

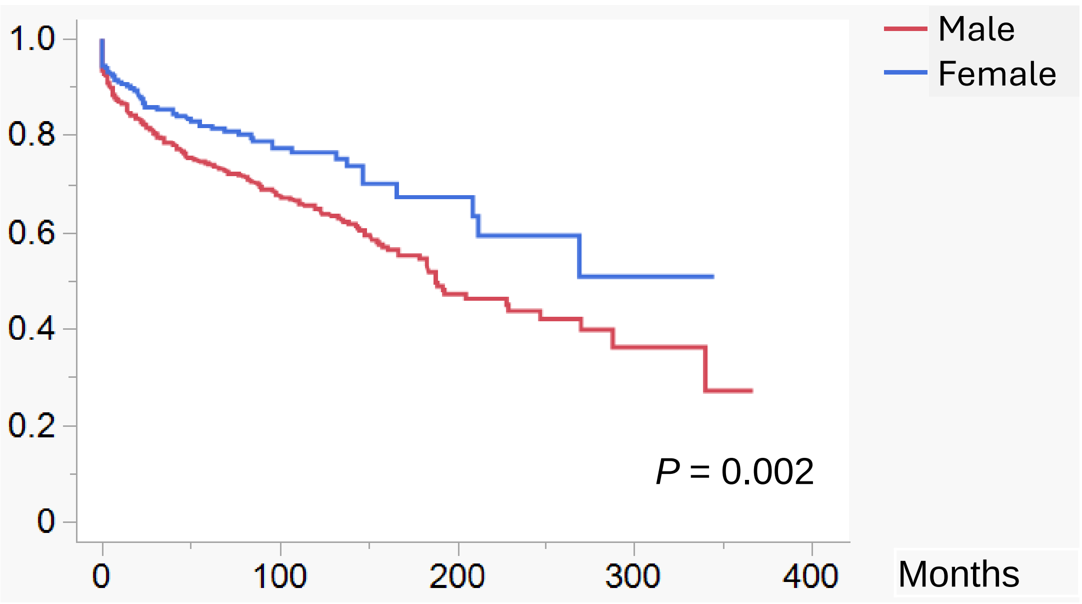


No. at risk:

| | | | | | |
|---|---|---|---|---|---|
| Male | 713 | 229 | 53 | 8 | 0 |
| Female | 247 | 98 | 19 | 2 | 0 |

**(M) OS according to surgical procedure (*n* = 960)**

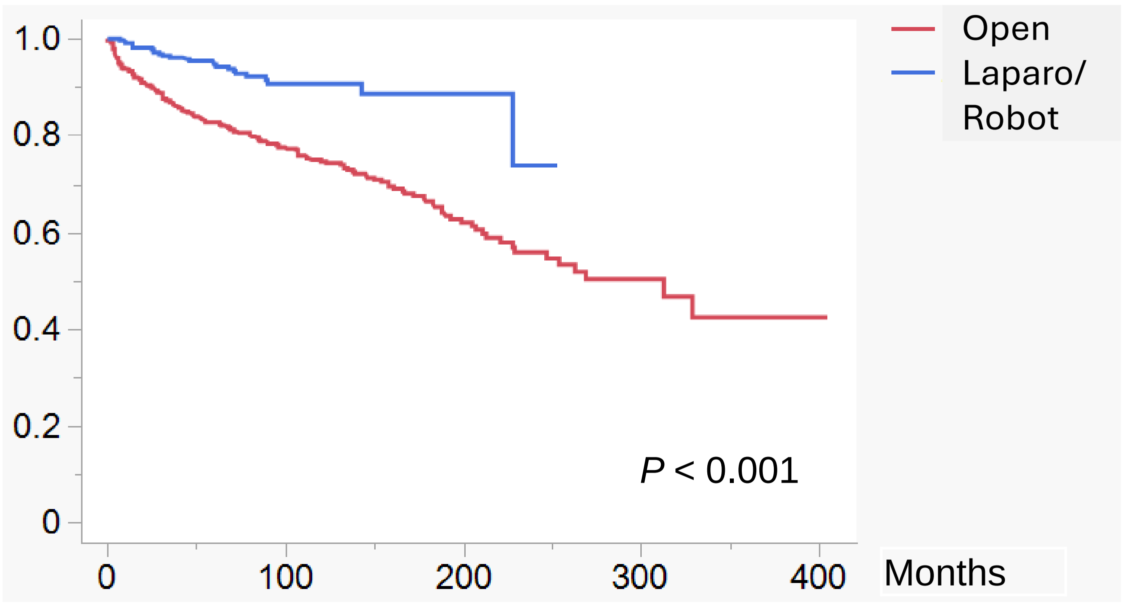


No. at risk:

| | | | | | |
|---|---|---|---|---|---|
| Open | 626 | 289 | 86 | 15 | 1 |
| Laparo/Robot | 334 | 101 | 10 | 0 | 0 |

**(N) CSS according to surgical procedure (*n* = 960)**

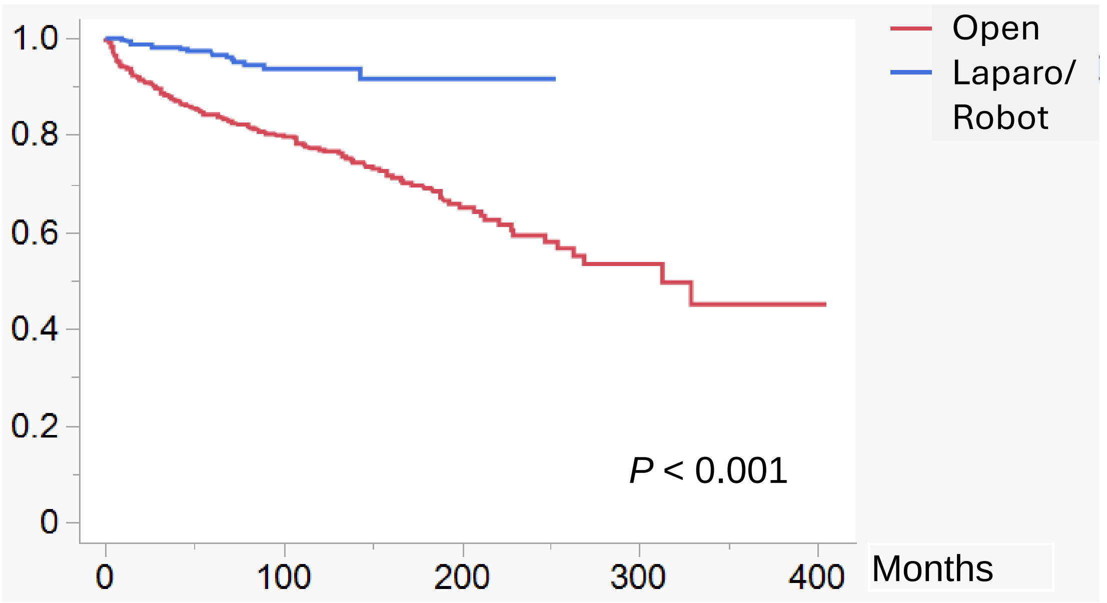


No. at risk:

| | | | | | |
|---|---|---|---|---|---|
| Open | 626 | 289 | 86 | 15 | 1 |
| Laparo/Robot | 334 | 101 | 10 | 0 | 0 |

**(O) RFS according to surgical procedure (*n* = 960)**

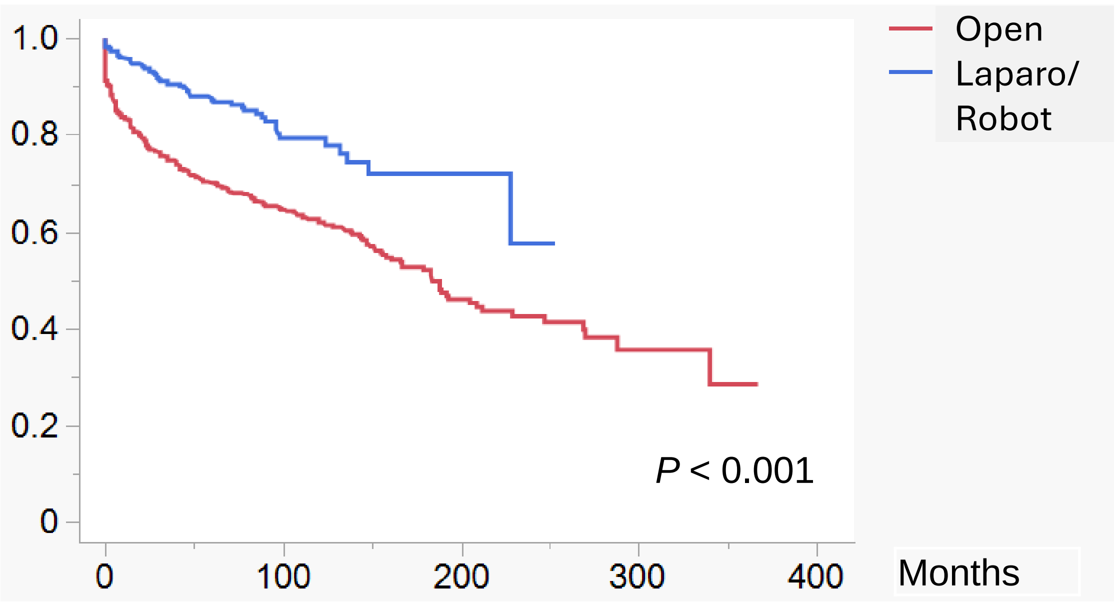


No. at risk:

| | | | | | |
|---|---|---|---|---|---|
| Open | 626 | 243 | 63 | 10 | 0 |
| Laparo/Robot | 334 | 84 | 9 | 0 | 0 |

**(P) OS according to nephrectomy type (*n* = 960)**

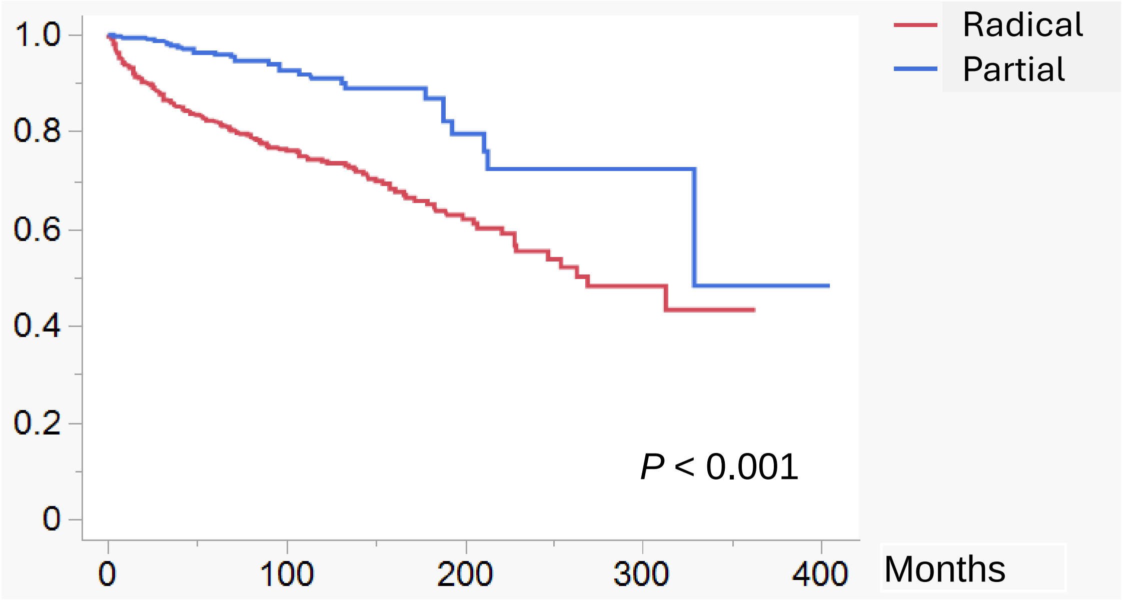


No. at risk:

| | | | | | |
|---|---|---|---|---|---|
| Radical | 625 | 260 | 69 | 11 | 0 |
| Partial | 335 | 130 | 27 | 4 | 1 |

**(Q) CSS according to nephrectomy type (*n* = 960)**

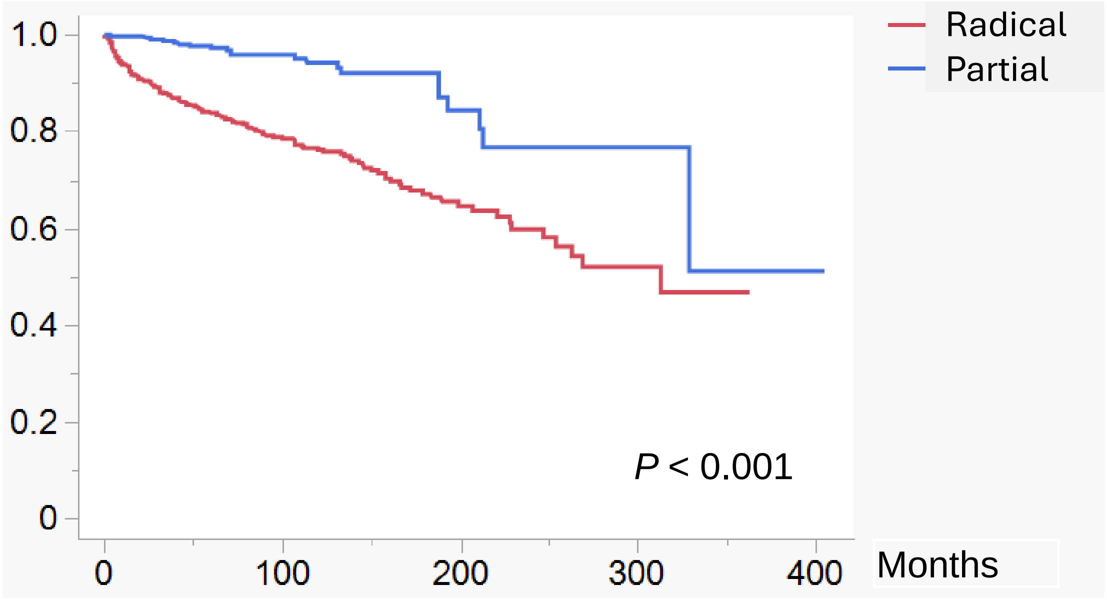


No. at risk:

| | | | | | |
|---|---|---|---|---|---|
| Radical | 625 | 260 | 69 | 11 | 0 |
| Partial | 335 | 130 | 27 | 4 | 1 |

**(R) RFS according to nephrectomy type (*n* = 960)**

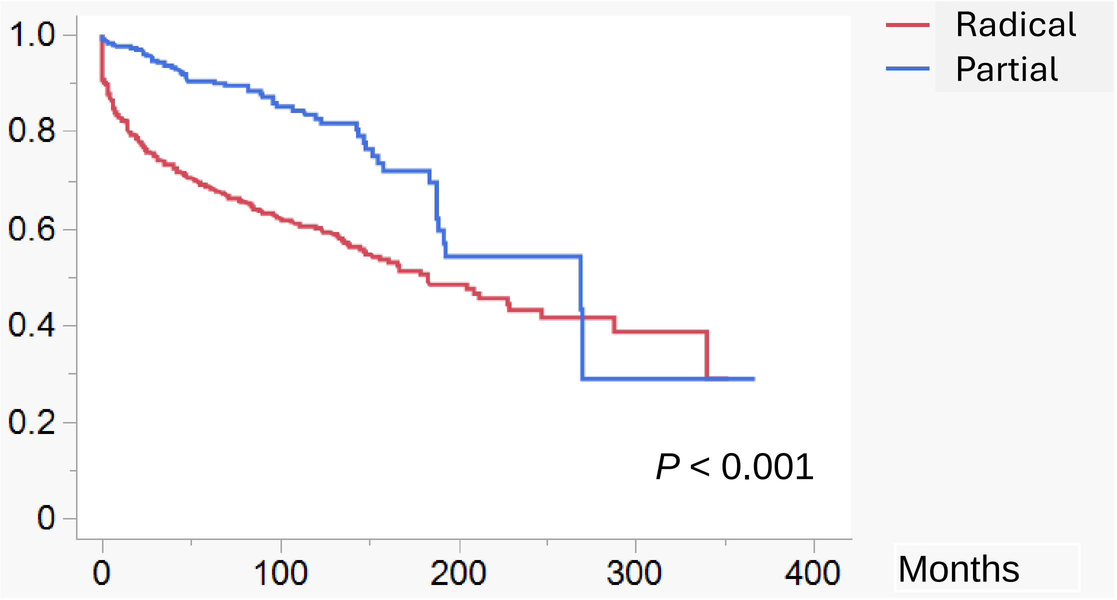


No. at risk:

| | | | | | |
|---|---|---|---|---|---|
| Radical | 625 | 208 | 55 | 9 | 0 |
| Partial | 335 | 119 | 17 | 1 | 0 |

**(S) OS according to histological type (*n* = 960)**

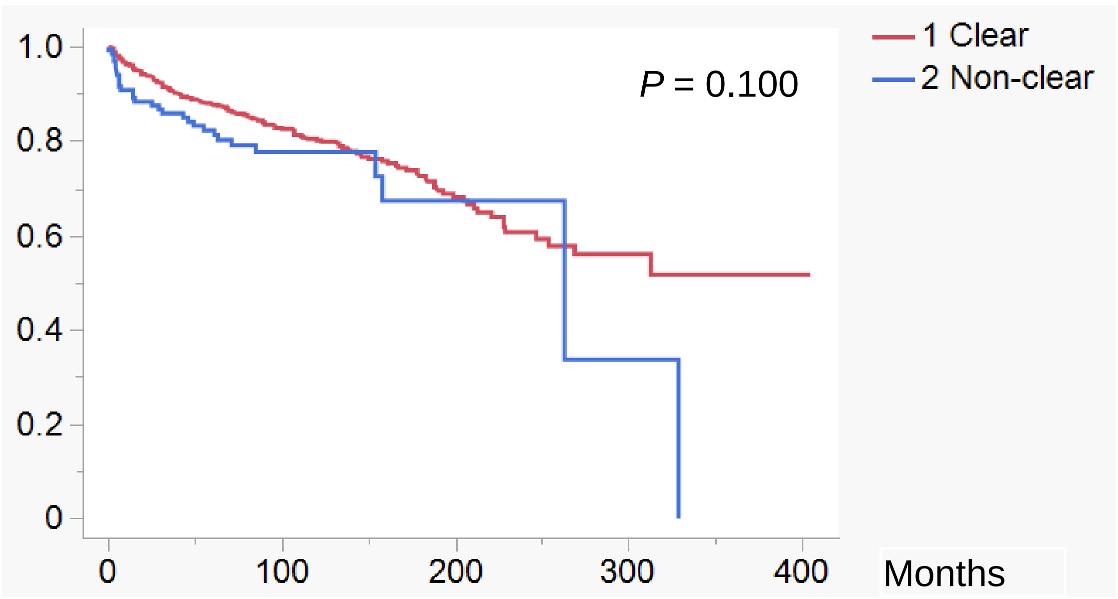


No. at risk:

| | | | | | |
|---|---|---|---|---|---|
| Clear | 825 | 344 | 92 | 14 | 1 |
| Non-clear | 135 | 46 | 4 | 1 | 0 |

**(T) CSS according to histological type (*n* = 960)**

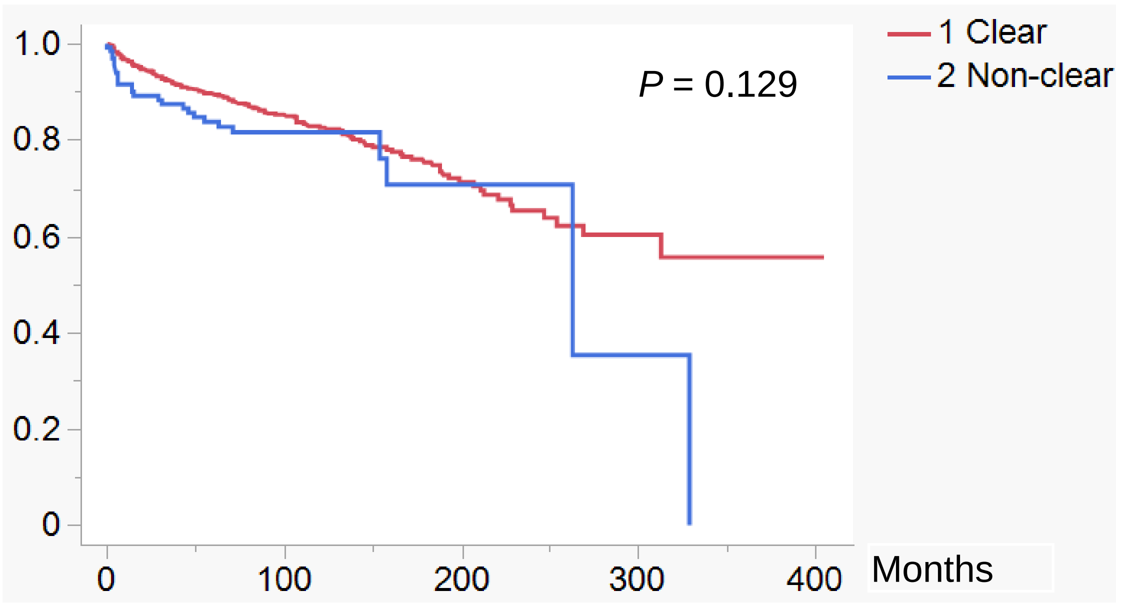


No. at risk:

| | | | | | |
|---|---|---|---|---|---|
| Clear | 825 | 344 | 92 | 14 | 1 |
| Non-clear | 135 | 46 | 4 | 1 | 0 |

**(U) RFS according to histological type (*n* = 960)**

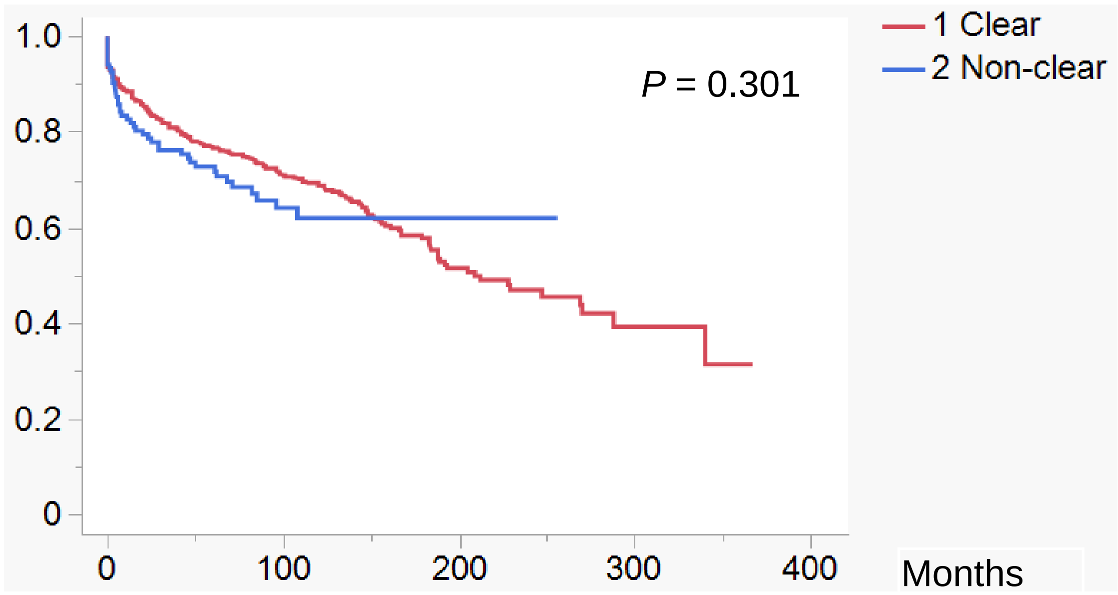


No. at risk:

| | | | | | |
|---|---|---|---|---|---|
| Clear | 825 | 290 | 70 | 10 | 0 |
| Non-clear | 135 | 37 | 2 | 0 | 0 |
