# Supplementary material for: Survival improvement over time in renal cell carcinoma treated with nephrectomy: A longitudinal propensity score‐matched study
Source: Int J Urol. 2024 Oct 28;32(2):145–50. doi: 10.1111/iju.15610 (PMC11803181; doi:10.1111/iju.15610)
Supplement: Supplementary file 2 — Figure S2. [file IJU-32-145-s006.pdf]

(A) OS according to the era in pStage I (n = 684)

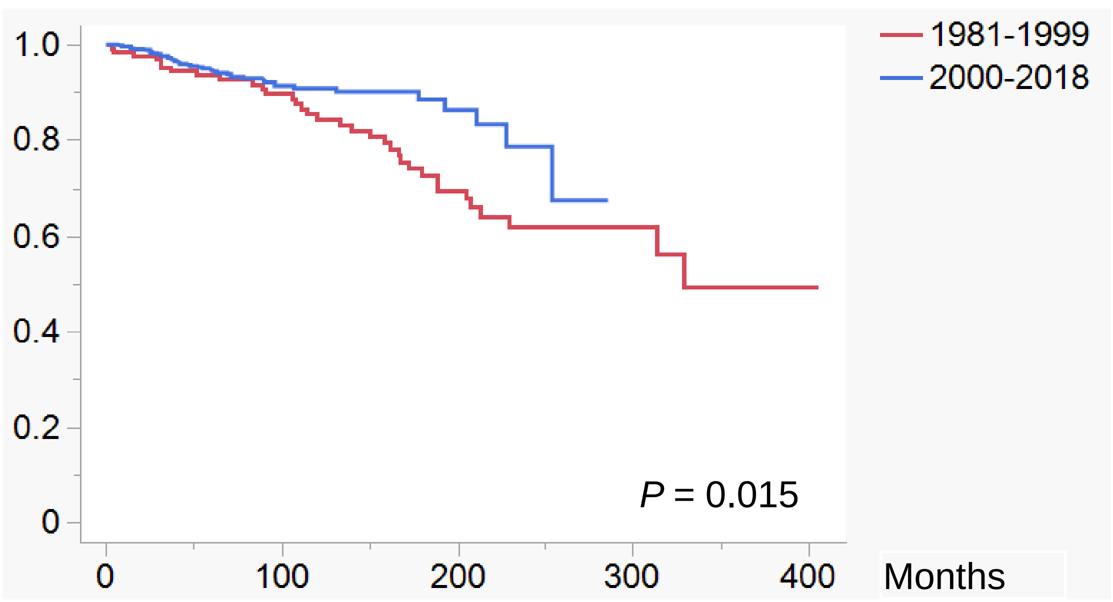

No. at risk:

1981–1999	139	88	41	12	1
2000–2018	545	208	34	0	0

(B) CSS according to the era in pStage I (n = 684)

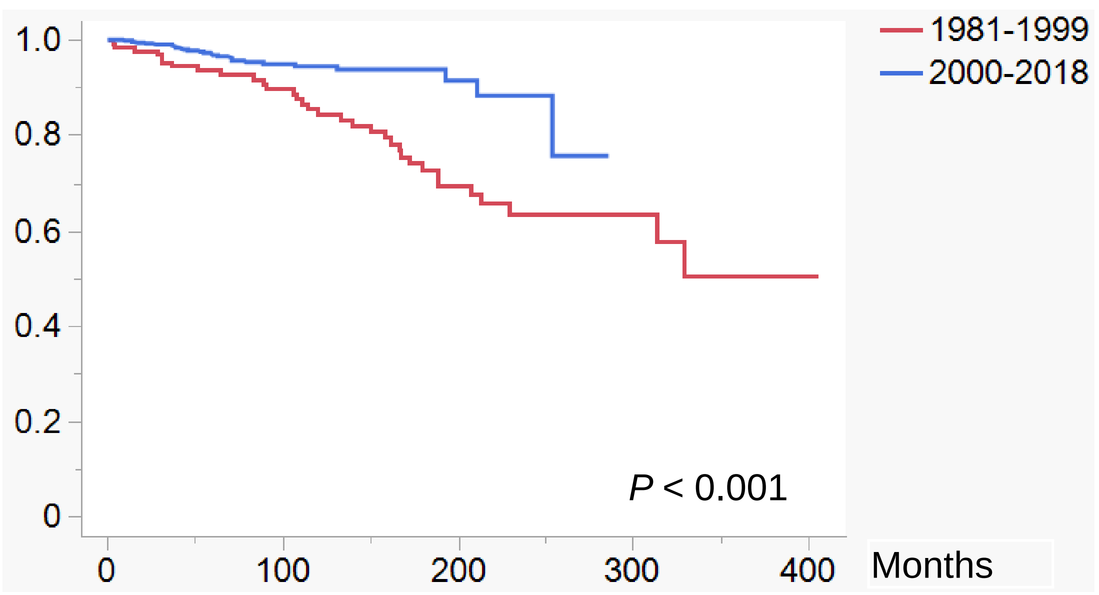

No. at risk:

1981–1999	139	88	41	12	1
2000–2018	545	208	34	0	0

(C) RFS according to the era in pStage I (n = 684)

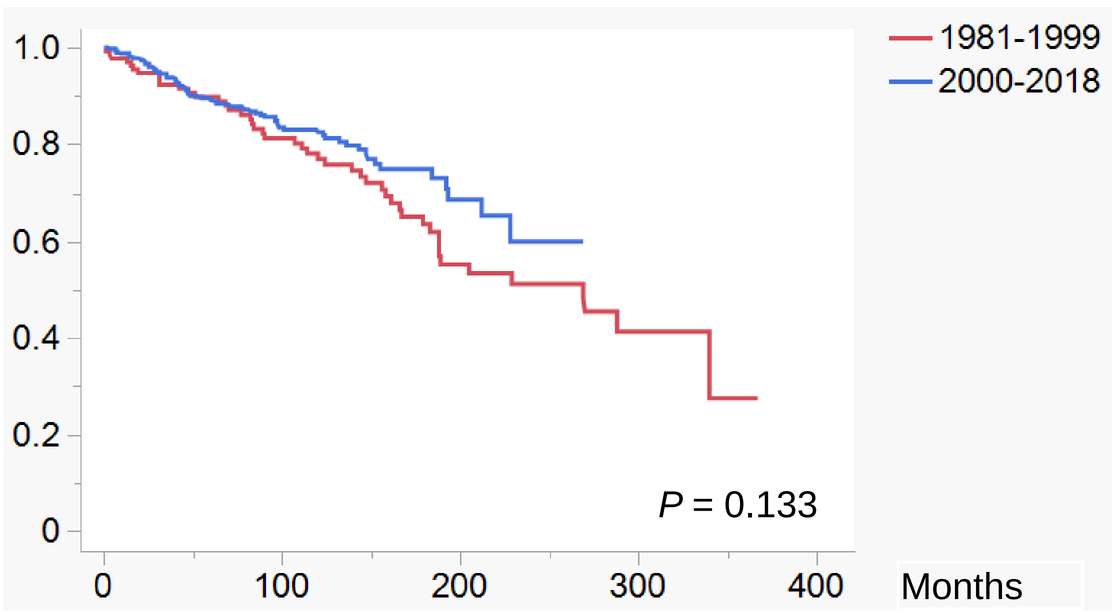

No. at risk:

1981–1999	139	80	32	7	0
2000–2018	545	185	25	0	0

(D) OS according to the era in pStage II (n = 77)

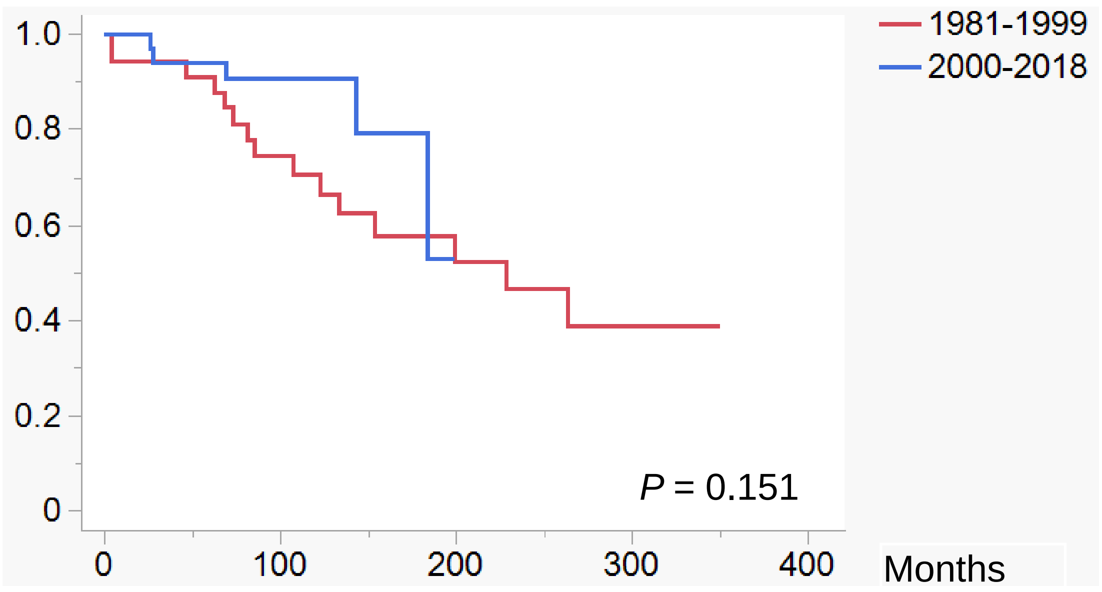

No. at risk:

1981–1999	36	21	10	2	0
2000–2018	41	20	0	0	0

(E) CSS according to the era in pStage II (n = 77)

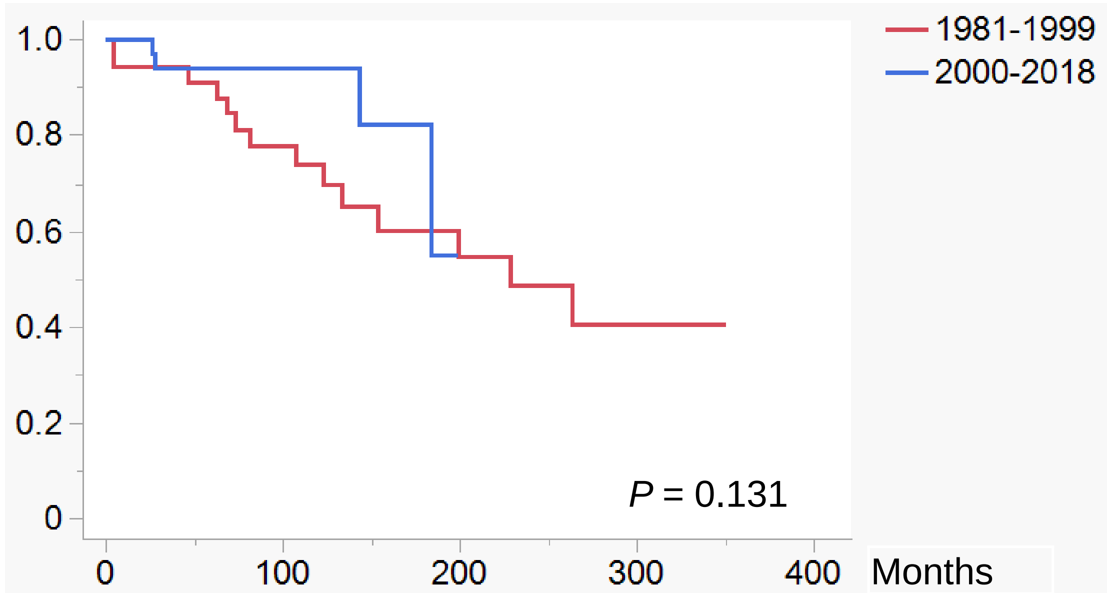

No. at risk:

1981–1999	36	21	10	2	0
2000–2018	41	20	0	0	0

(F) RFS according to the era in pStage II (n = 77)

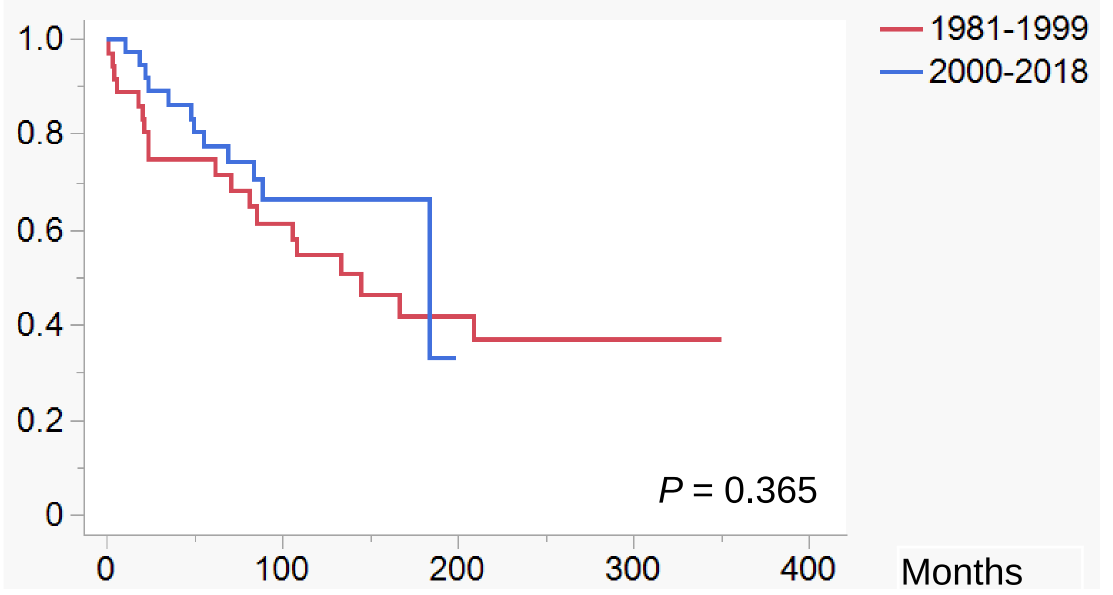

No. at risk:

1981–1999	36	18	9	2	0
2000–2018	41	14	0	0	0

(G) OS according to the era in pStage III (*n* = 130)

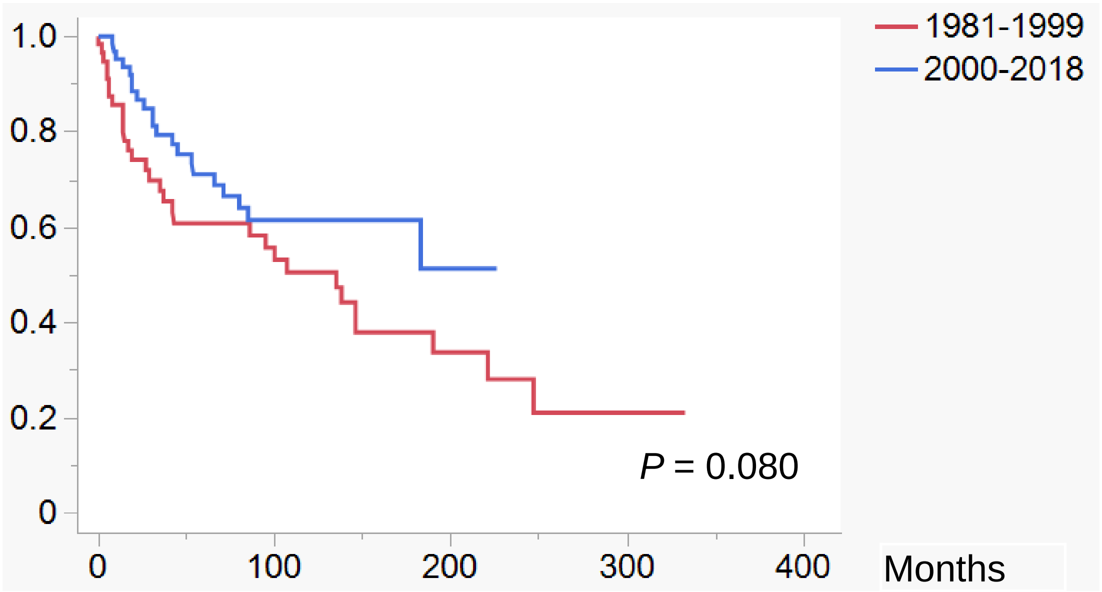

No. at risk:

1981–1999	60	22	7	1	0
2000–2018	70	21	1	0	0

(H) CSS according to the era in pStage III (*n* = 130)

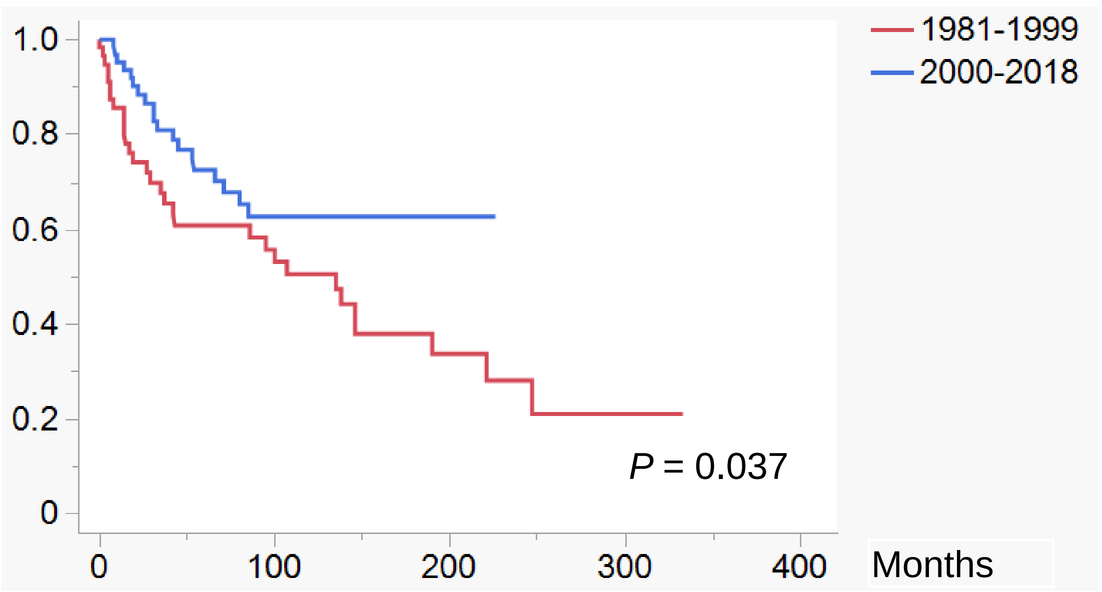

No. at risk:

1981–1999	60	22	7	1	0
2000–2018	70	21	1	0	0

(I) RFS according to the era in pStage III (*n* = 130)

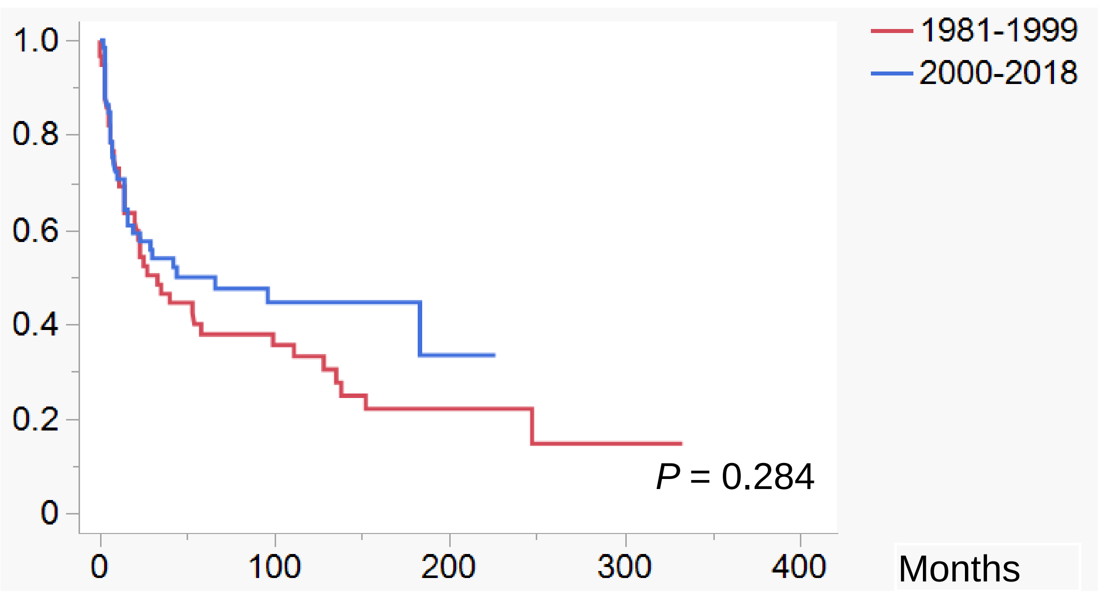

No. at risk:

1981–1999	60	16	5	1	0
2000–2018	70	13	1	0	0

(J) OS according to the era in pStage IV (*n* = 69)

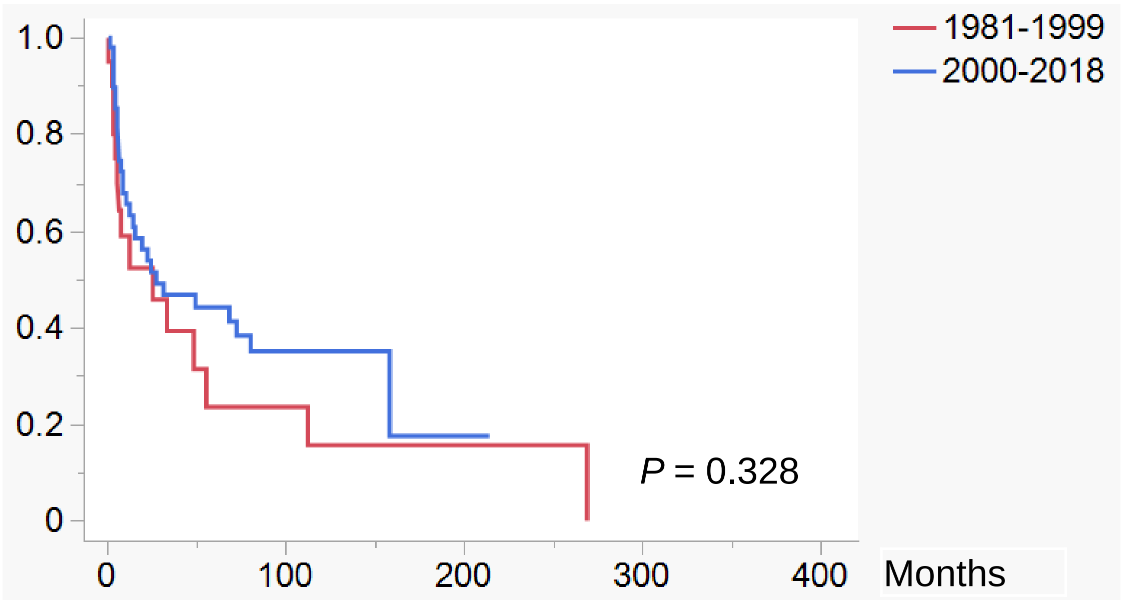

No. at risk:

1981–1999	20	3	2	0	0
2000–2018	49	7	1	0	0

(K) CSS according to the era in pStage IV (*n* = 69)

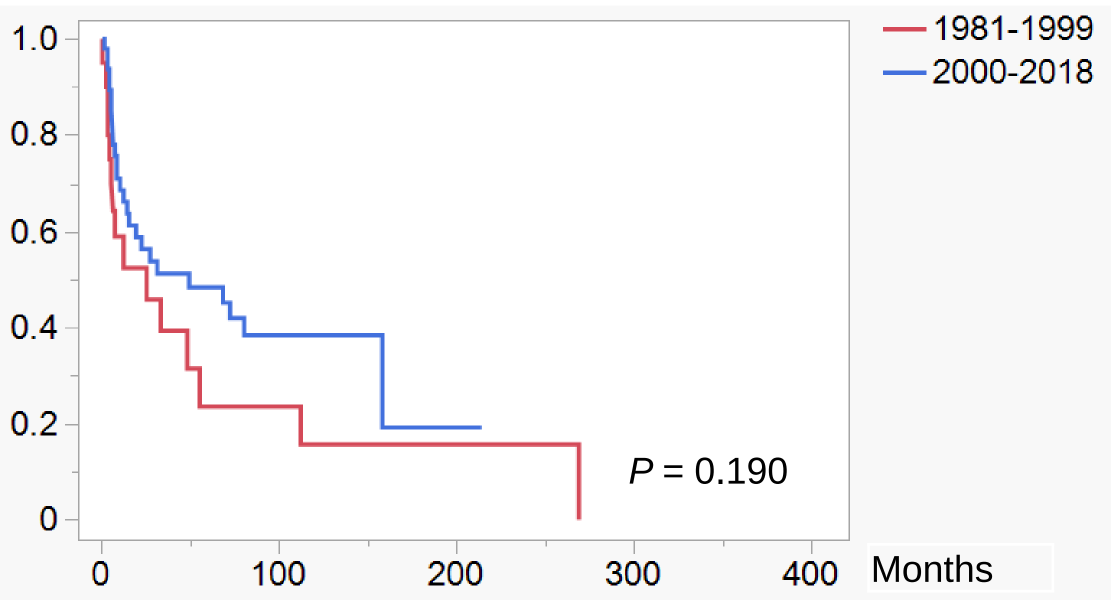

No. at risk:

1981–1999	20	3	2	0	0
2000–2018	49	7	1	0	0

(L) RFS according to the era in pStage IV (*n* = 69)

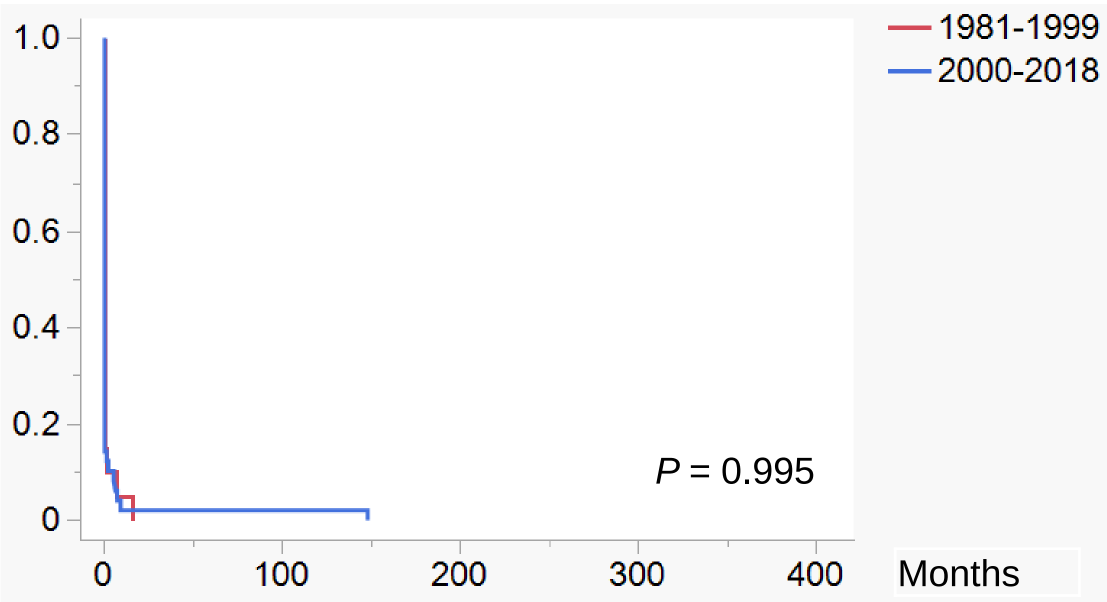

No. at risk:

1981–1999	20	0	0	0	0
2000–2018	49	1	0	0	0
