# Supplementary material for: Survival improvement over time in renal cell carcinoma treated with nephrectomy: A longitudinal propensity score‐matched study
Source: Int J Urol. 2024 Oct 28;32(2):145–50. doi: 10.1111/iju.15610 (PMC11803181; doi:10.1111/iju.15610)
Supplement: Supplementary file 3 — Figure S3. [file IJU-32-145-s003.pdf]

(A) OS according to the detailed era (*n* = 466)

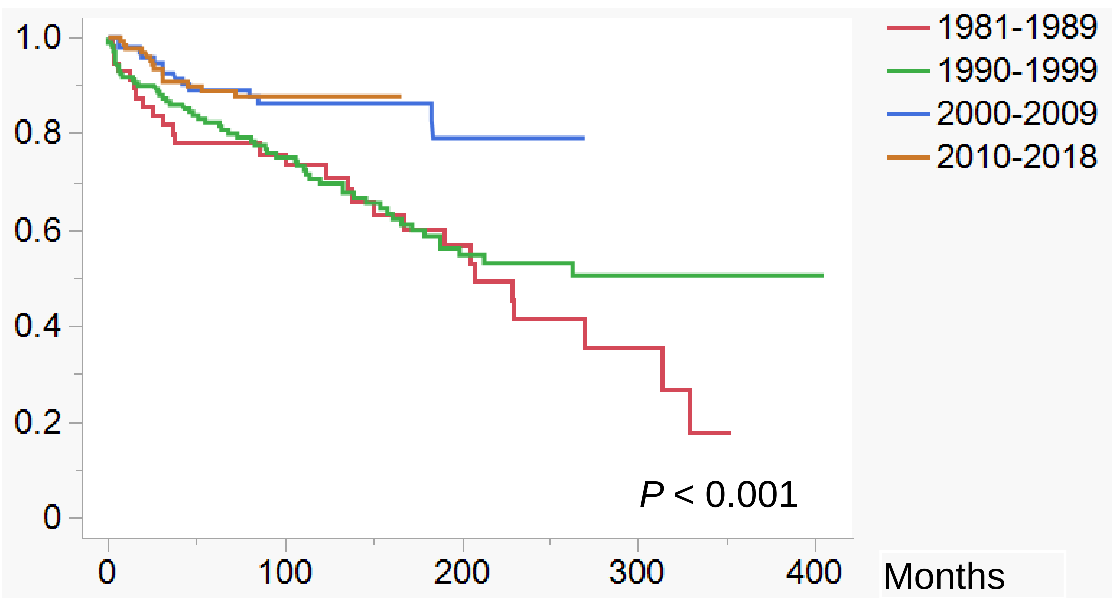

No. at risk:

1981–1989	59	34	16	4	0
1990–1999	174	87	38	10	1
2000–2009	99	55	10	0	0
2010–2018	134	36	0	0	0

(B) CSS according to the detailed era (*n* = 466)

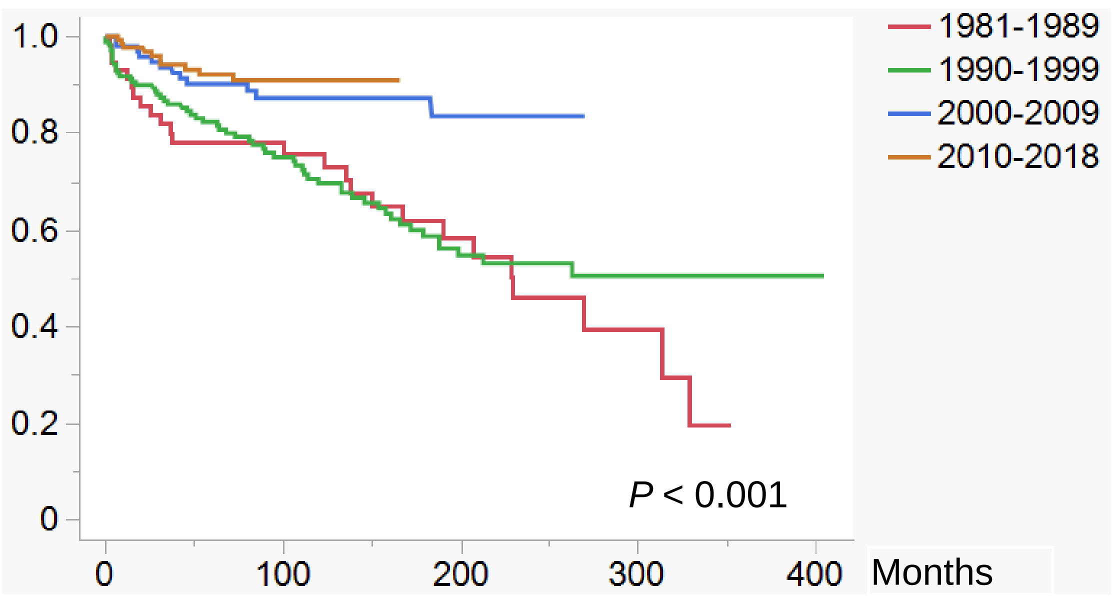

No. at risk:

1981–1989	59	34	16	4	0
1990–1999	174	87	38	10	1
2000–2009	99	55	10	0	0
2010–2018	134	36	0	0	0

(C) RFS according to the detailed era (*n* = 466)

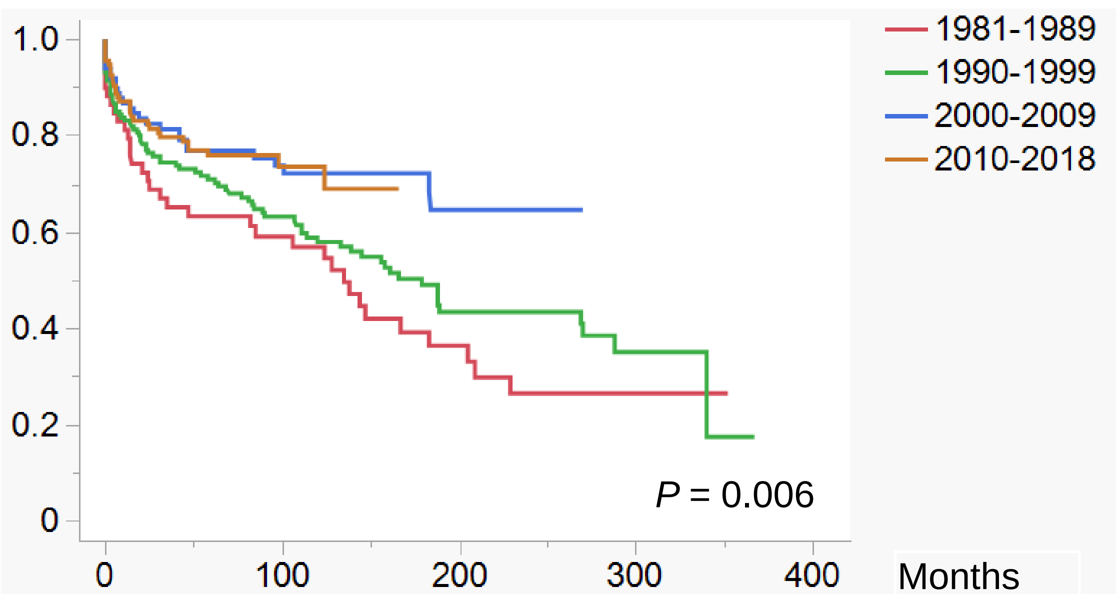

No. at risk:

1981–1989	59	28	12	2	0
1990–1999	174	76	29	7	0
2000–2009	99	46	9	0	0
2010–2018	134	29	0	0	0

(D) OS according to pStage (*n* = 466)

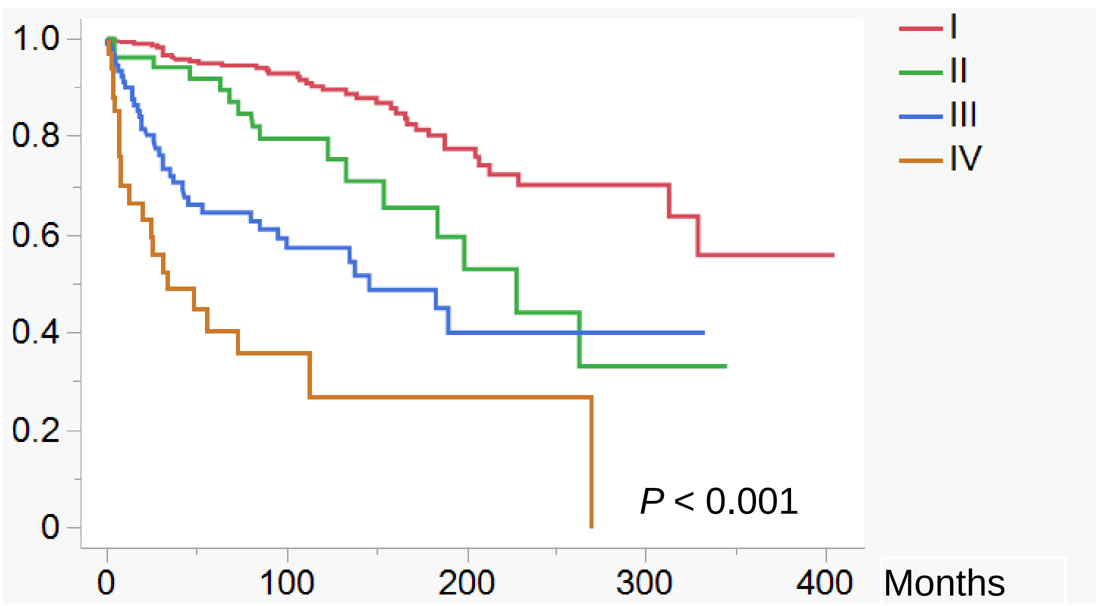

No. at risk:

I	285	151	49	12	1
II	52	25	7	1	0
III	95	31	6	1	0
IV	34	5	2	0	0

(E) CSS according to pStage (*n* = 466)

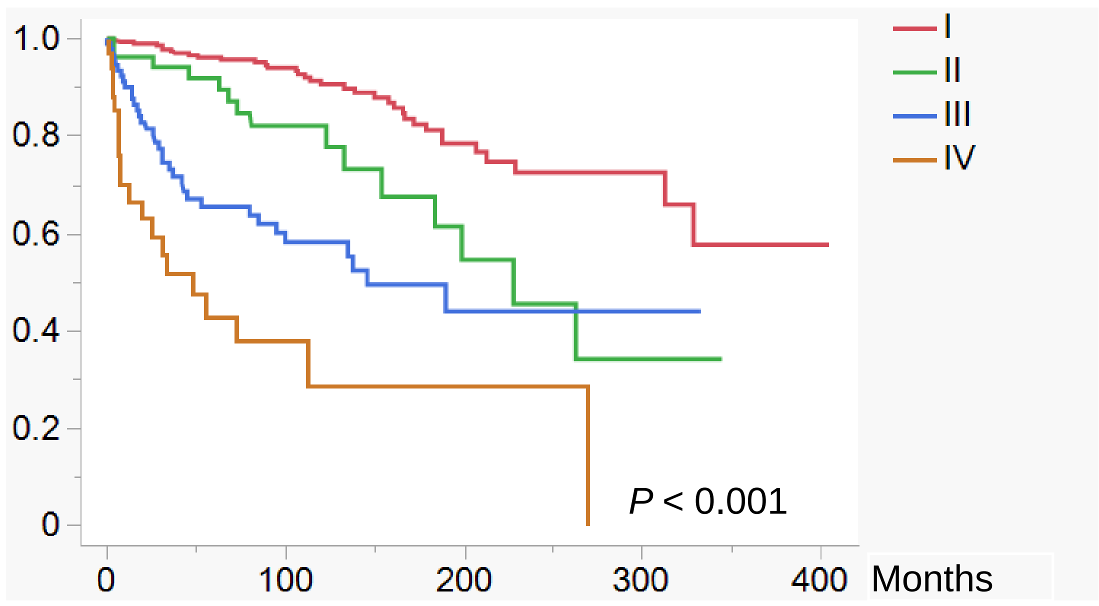

No. at risk:

I	285	151	49	12	1
II	52	25	7	1	0
III	95	31	6	1	0
IV	34	5	2	0	0

(F) RFS according to pStage (*n* = 466)

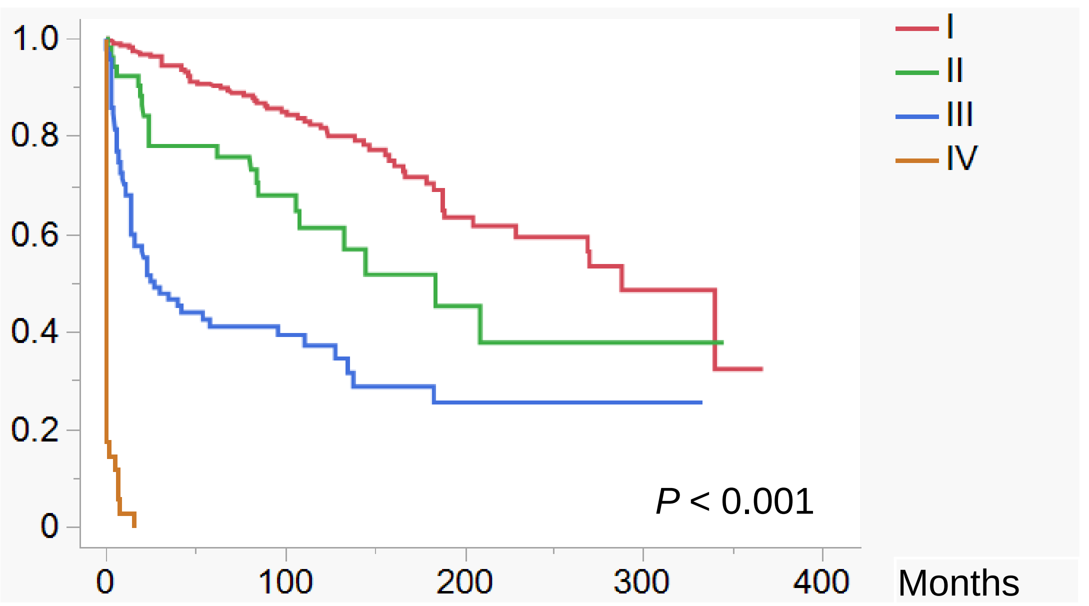

No. at risk:

I	285	136	39	7	0
II	52	22	6	1	0
III	95	21	5	1	0
IV	34	0	0	0	0

(G) OS according to age (*n* = 466)

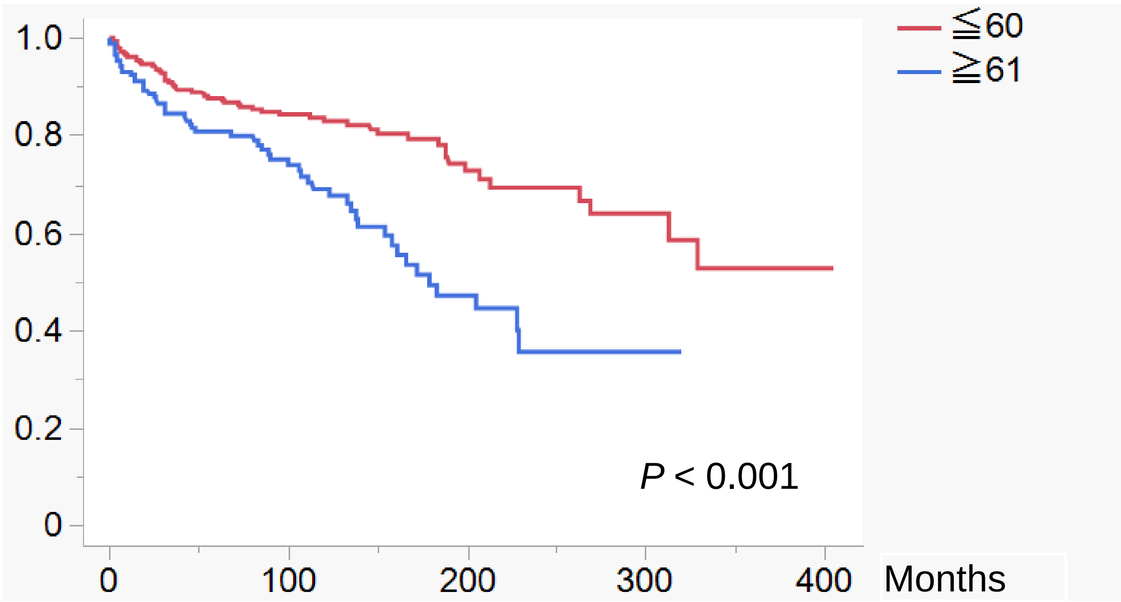

No. at risk:

≦60	290	145	46	12	1
≧61	176	67	18	2	0

(H) CSS according to age (*n* = 466)

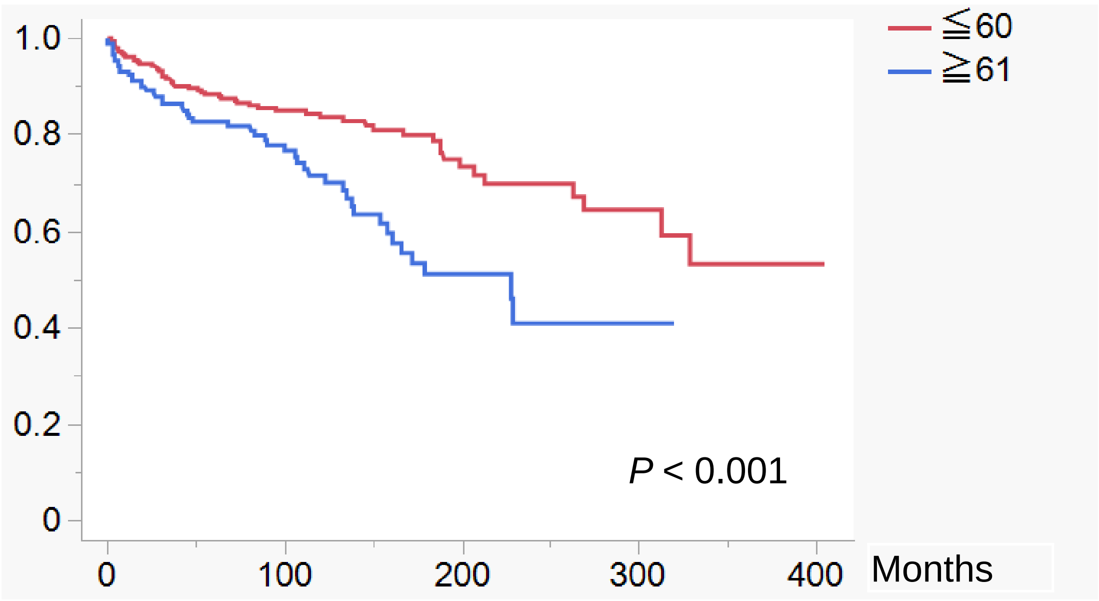

No. at risk:

≦60	290	145	46	12	1
≧61	176	67	18	2	0

(I) RFS according to age (*n* = 466)

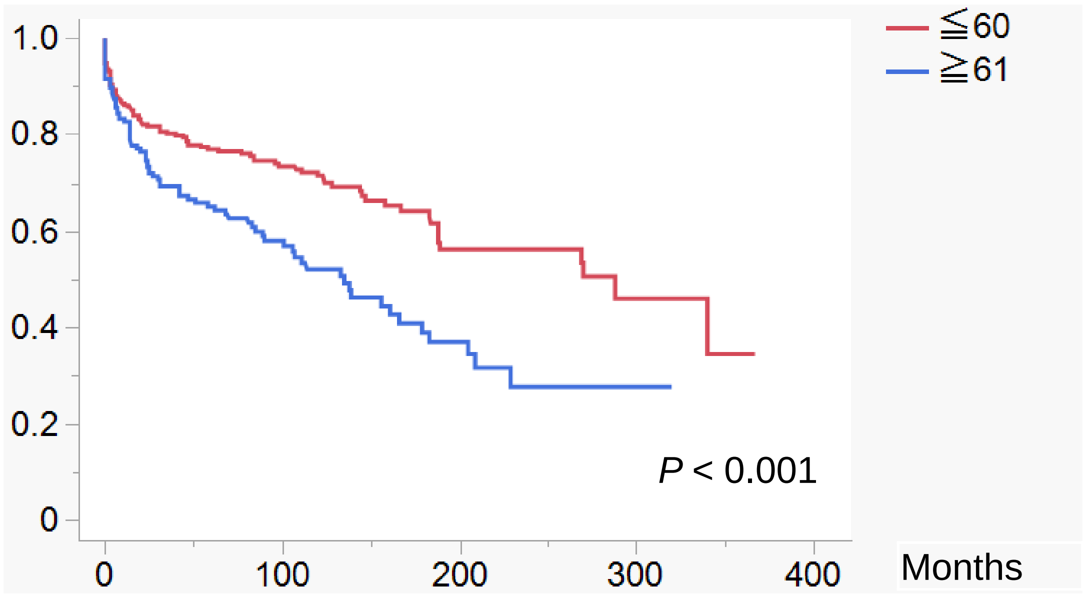

No. at risk:

≦60	290	126	35	7	0
≧61	176	53	15	2	0

(J) OS according to sex (*n* = 466)

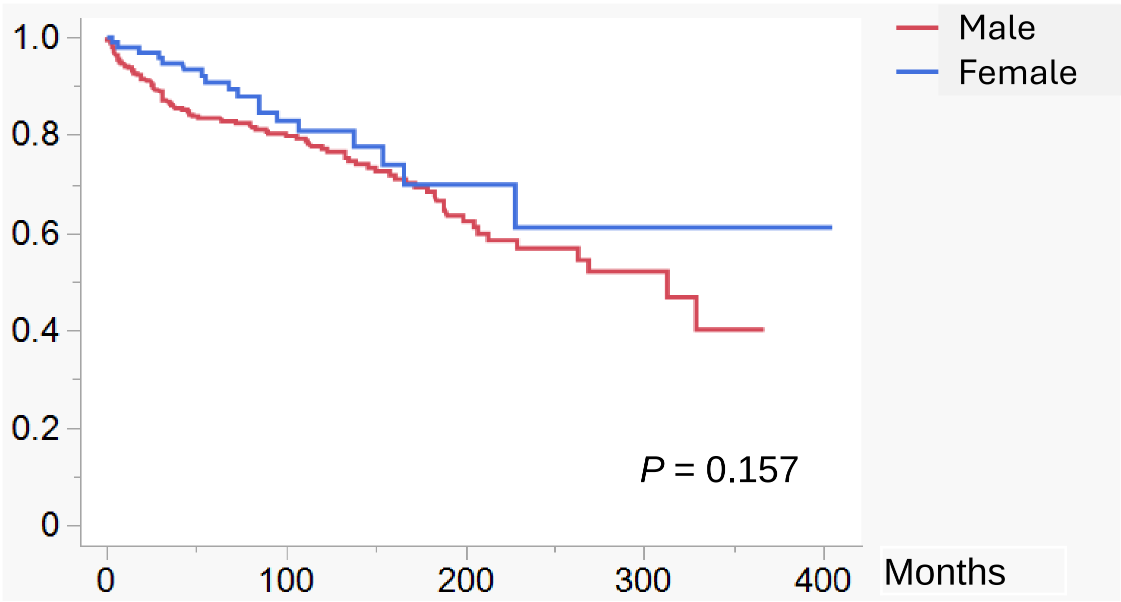

No. at risk:

Male	365	166	52	11	0
Female	101	46	12	3	1

(K) CSS according to sex (*n* = 466)

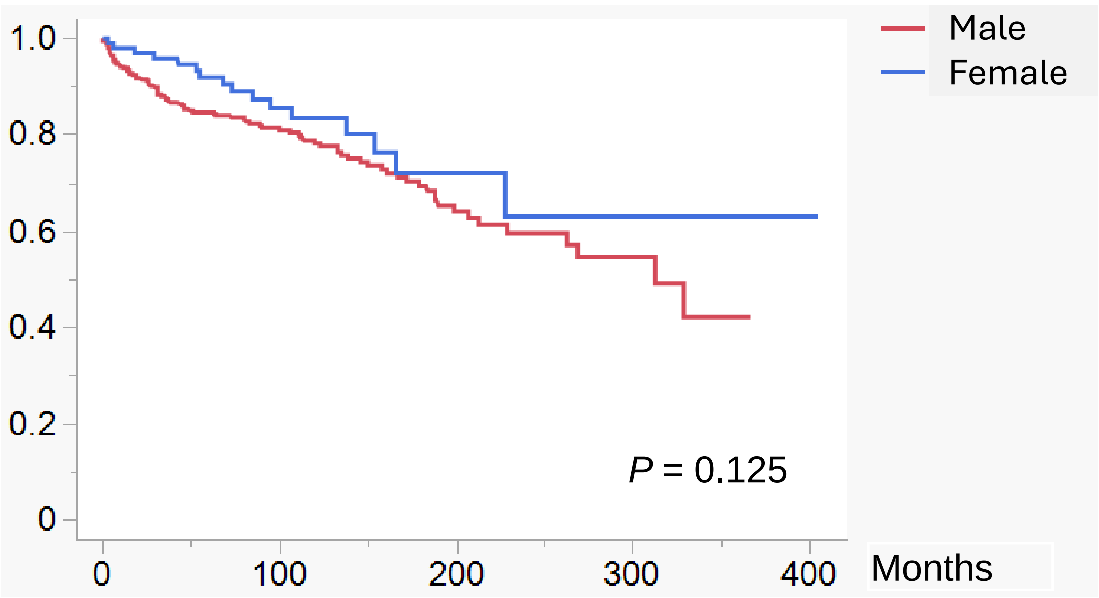

No. at risk:

Male	365	166	52	11	0
Female	101	46	12	3	1

(L) RFS according to sex (*n* = 466)

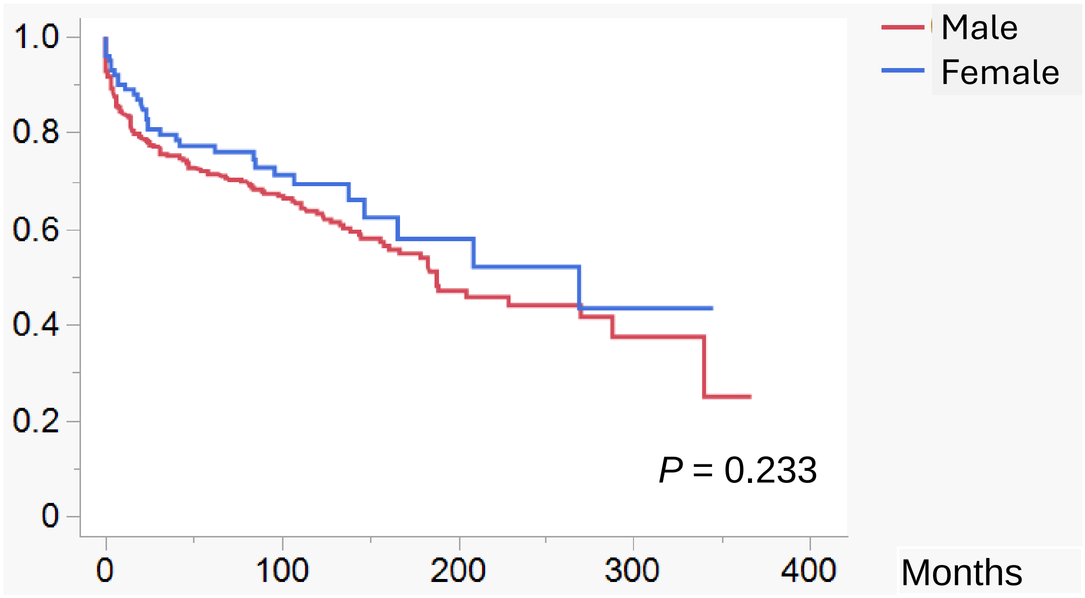

No. at risk:

Male	365	136	39	7	0
Female	101	43	11	2	0

(M) OS according to surgical procedure (*n* = 466)

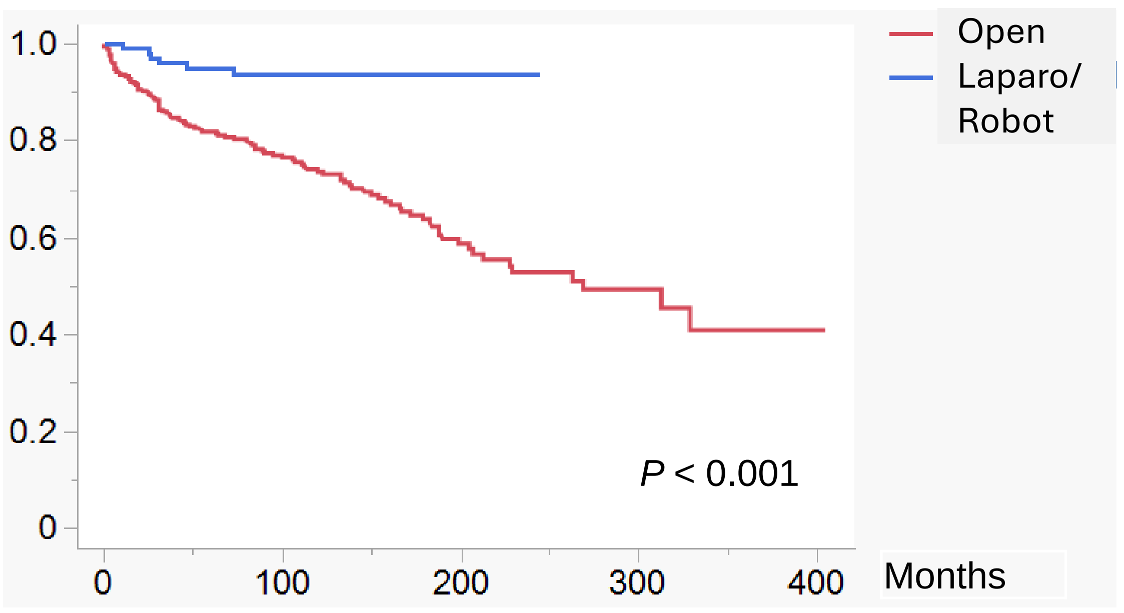

No. at risk:

Open	353	170	58	14	1
Laparo/Robot	113	42	6	0	0

(N) CSS according to surgical procedure (*n* = 466)

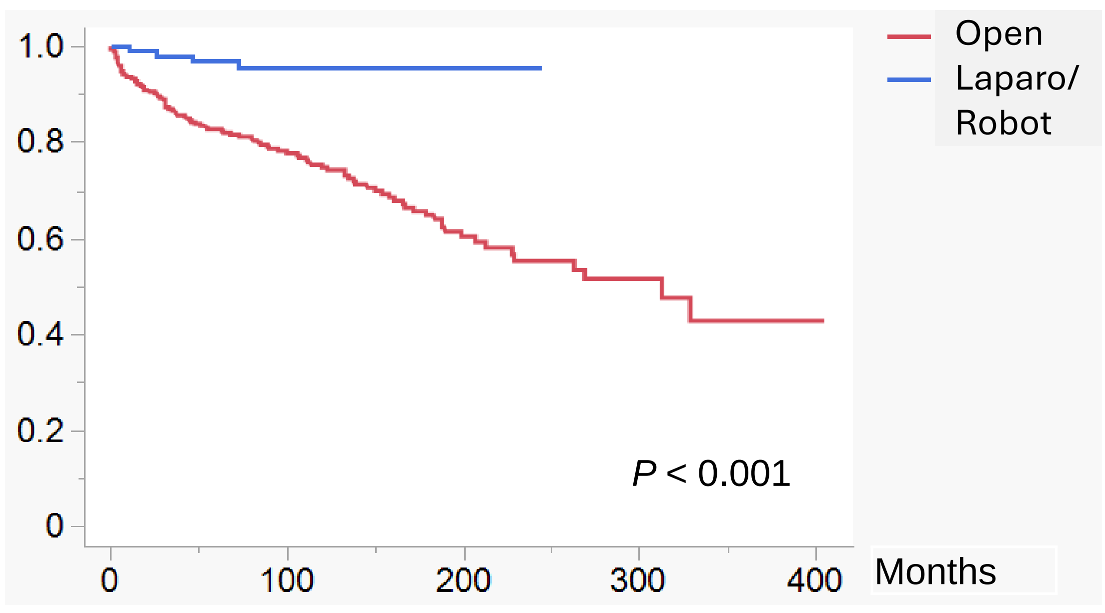

No. at risk:

Open	353	170	58	14	1
Laparo/Robot	113	42	6	0	0

(O) RFS according to surgical procedure (*n* = 466)

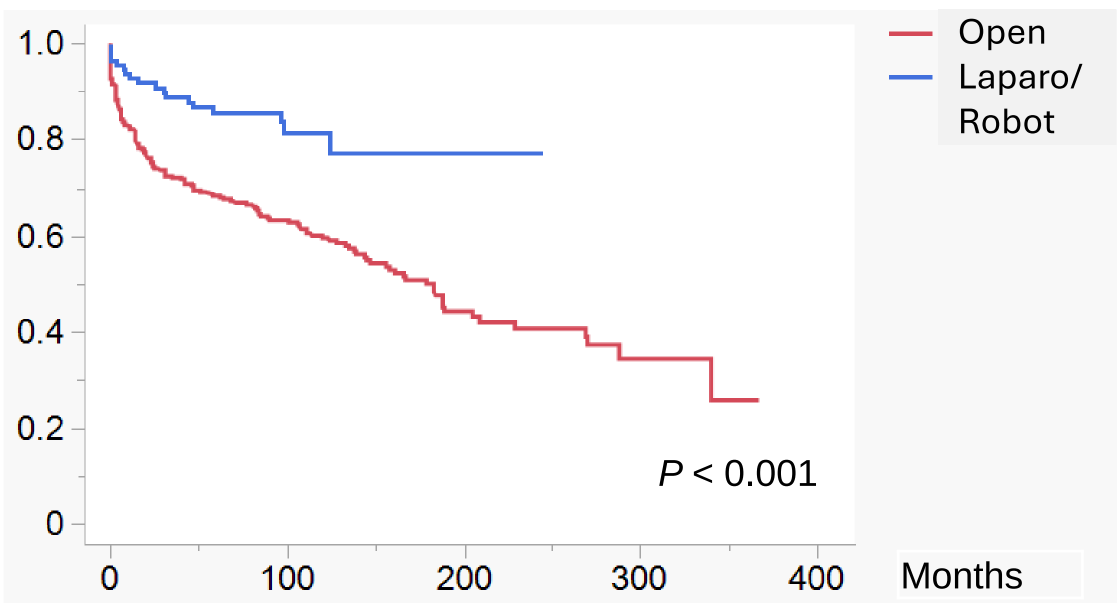

No. at risk:

Open	353	144	44	9	0
Laparo/Robot	113	35	6	0	0

(P) OS according to nephrectomy type (*n* = 466)

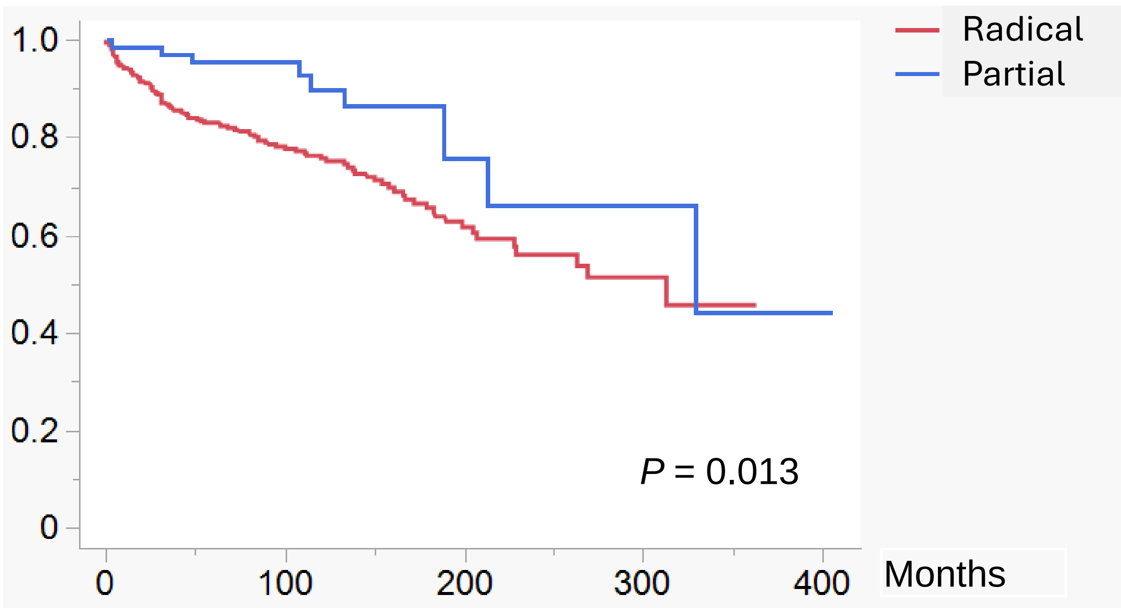

No. at risk:

Radical	387	177	54	10	0
Partial	79	35	10	4	1

(Q) CSS according to nephrectomy type (*n* = 466)

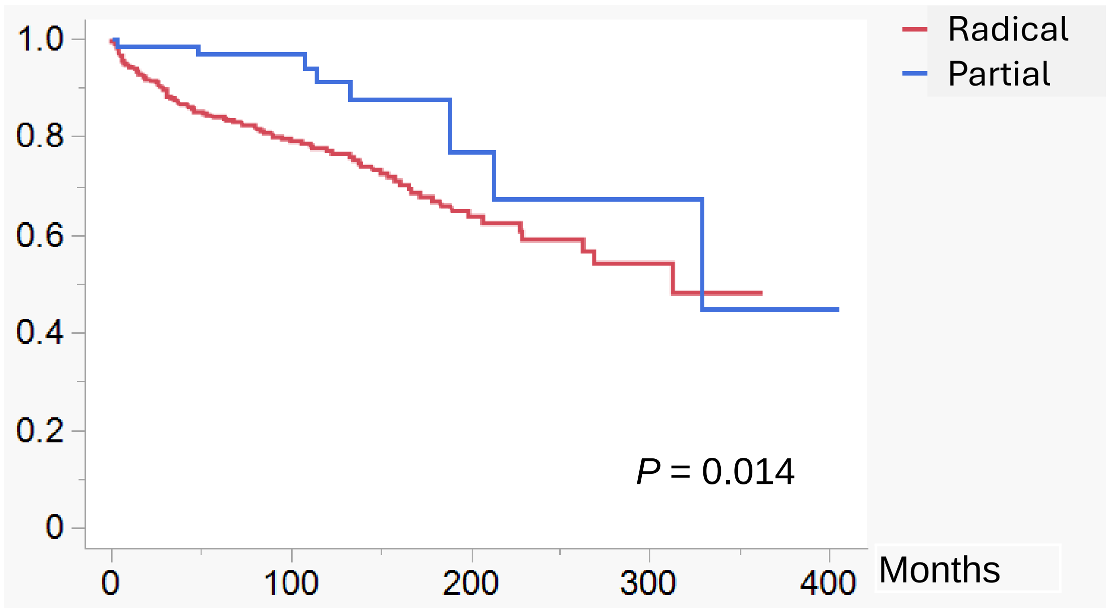

No. at risk:

Radical	387	177	54	10	0
Partial	79	35	10	4	1

(R) RFS according to nephrectomy type (*n* = 466)

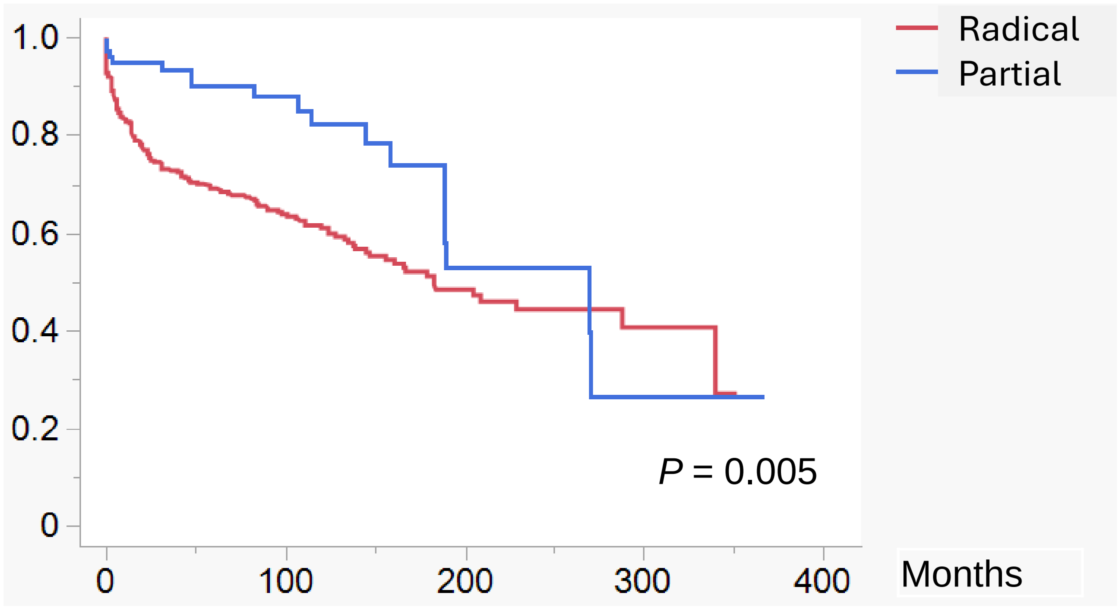

No. at risk:

Radical	387	147	43	8	0
Partial	79	32	7	1	0

(S) OS according to histological type (n = 466)

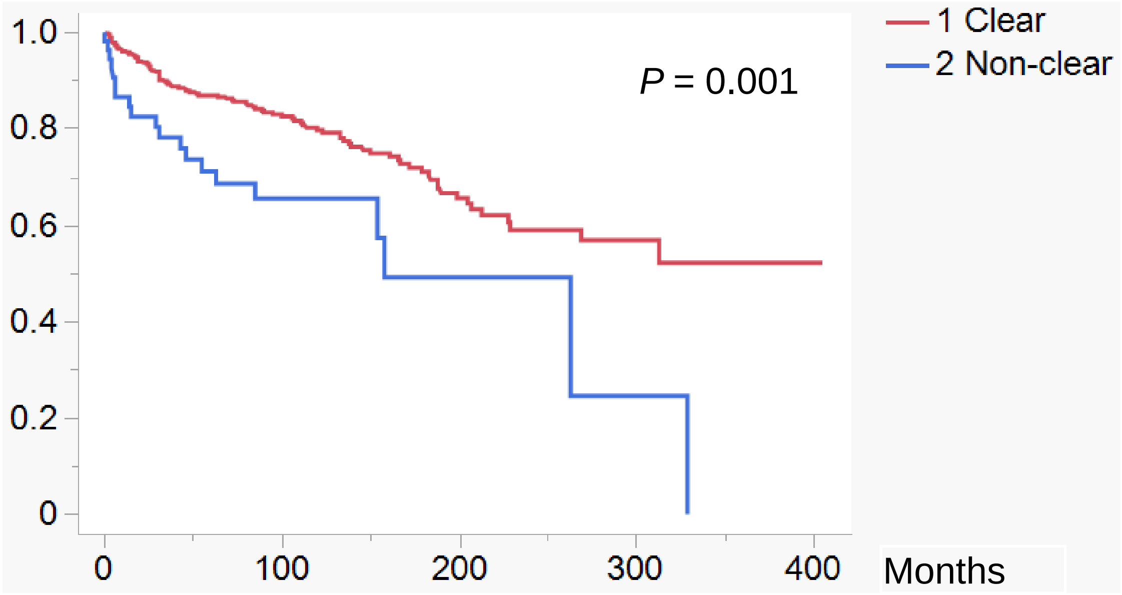

No. at risk:

Clear	413	194	61	13	1
Non-clear	53	18	3	1	0

(T) CSS according to histological type (n = 466)

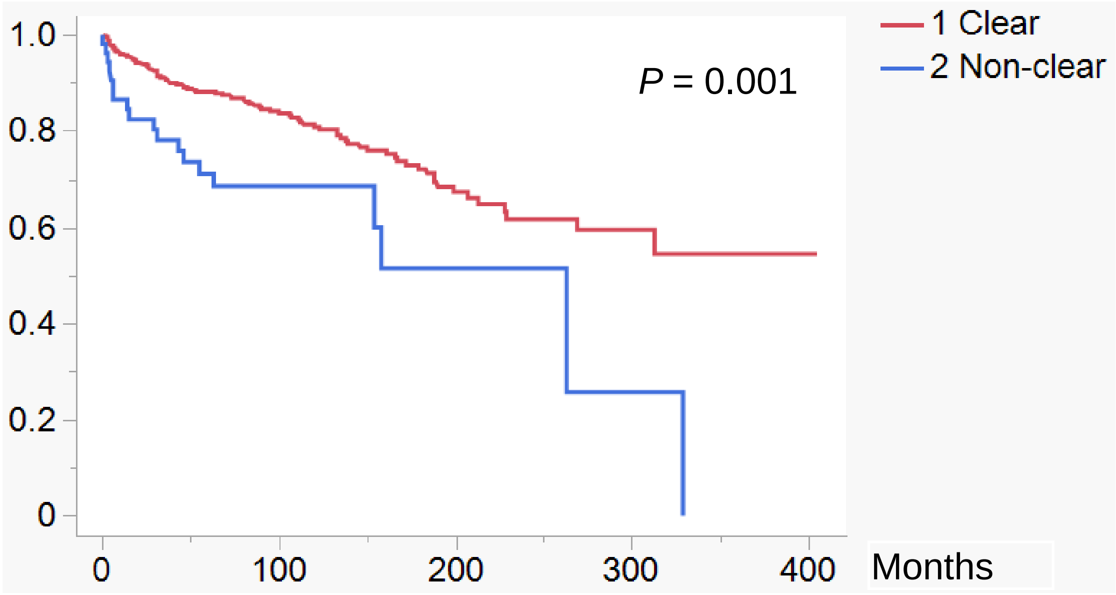

No. at risk:

Clear	413	194	61	13	1
Non-clear	53	18	3	1	0

(U) RFS according to histological type (n = 466)

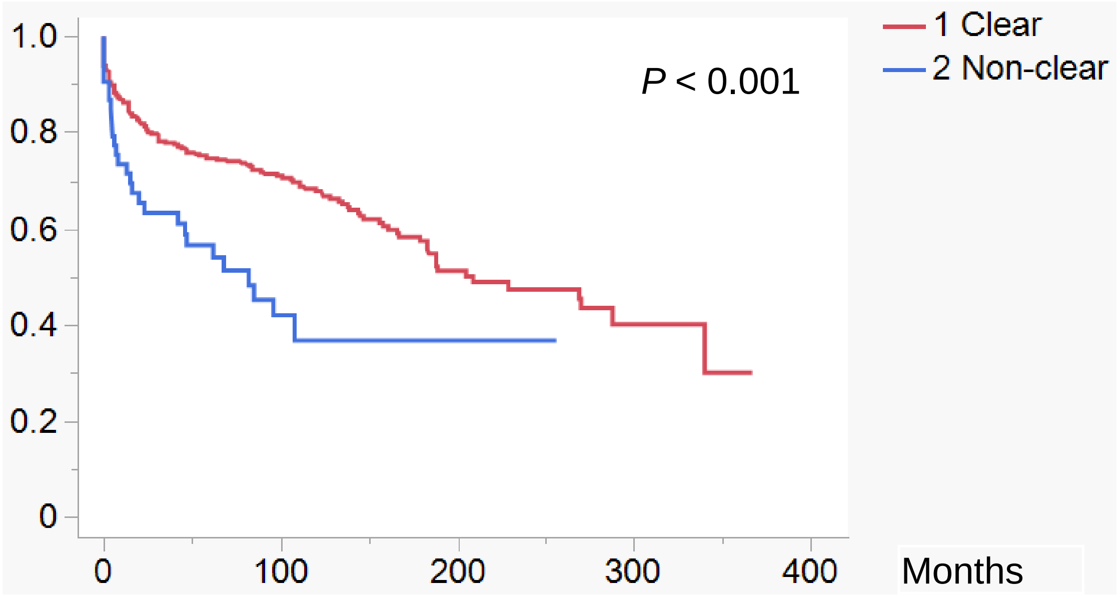

No. at risk:

Clear	413	168	49	9	0
Non-clear	53	11	1	0	0
