# Supplementary material for: Survival improvement over time in renal cell carcinoma treated with nephrectomy: A longitudinal propensity score‐matched study
Source: Int J Urol. 2024 Oct 28;32(2):145–50. doi: 10.1111/iju.15610 (PMC11803181; doi:10.1111/iju.15610)
Supplement: Supplementary file 4 — Figure S4. [file IJU-32-145-s005.pdf]

(A) OS according to the era in pStage I (n = 285) **(B) CSS according to the era in pStage I (n = 285)** **(C) RFS according to the era in pStage I (n = 285)**

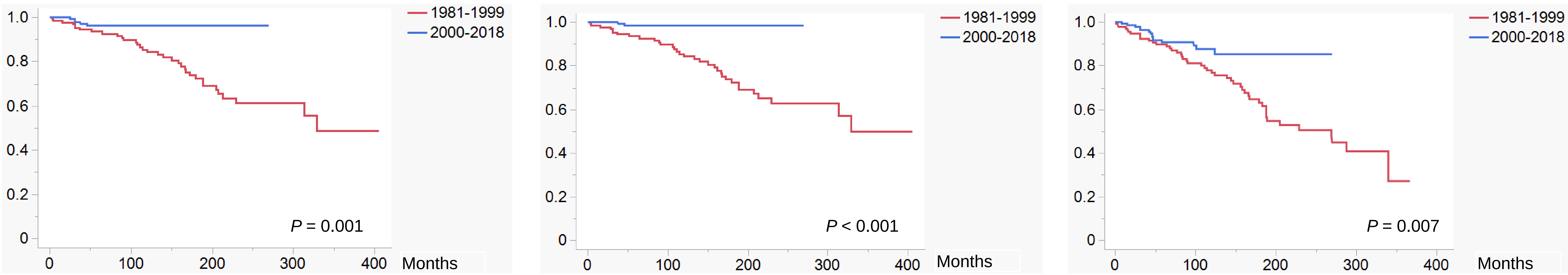

No. at risk: (A)

1981–1999	138	87	40	12	1
2000–2018	147	64	9	0	0

No. at risk: (B)

1981–1999	138	87	40	12	1
2000–2018	147	64	9	0	0

No. at risk: (C)

1981–1999	138	79	31	7	0
2000–2018	147	57	8	0	0

(D) OS according to the era in pStage II (n = 52) **(E) CSS according to the era in pStage II (n = 52)** **(F) RFS according to the era in pStage II (n = 52)**

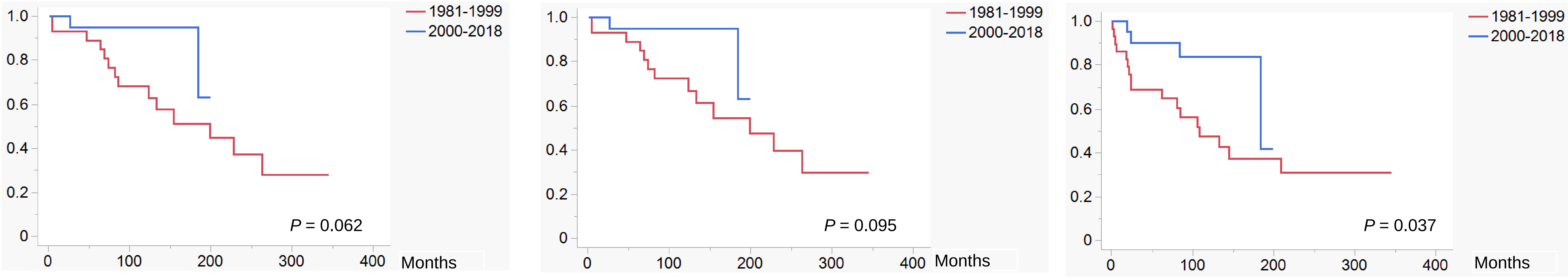

No. at risk: (D)

1981–1999	29	15	7	1	0
2000–2018	23	10	0	0	0

No. at risk: (E)

1981–1999	29	15	7	1	0
2000–2018	23	10	0	0	0

No. at risk: (F)

1981–1999	29	13	6	1	0
2000–2018	23	9	0	0	0

(G) OS according to the era in pStage III (*n* = 95)

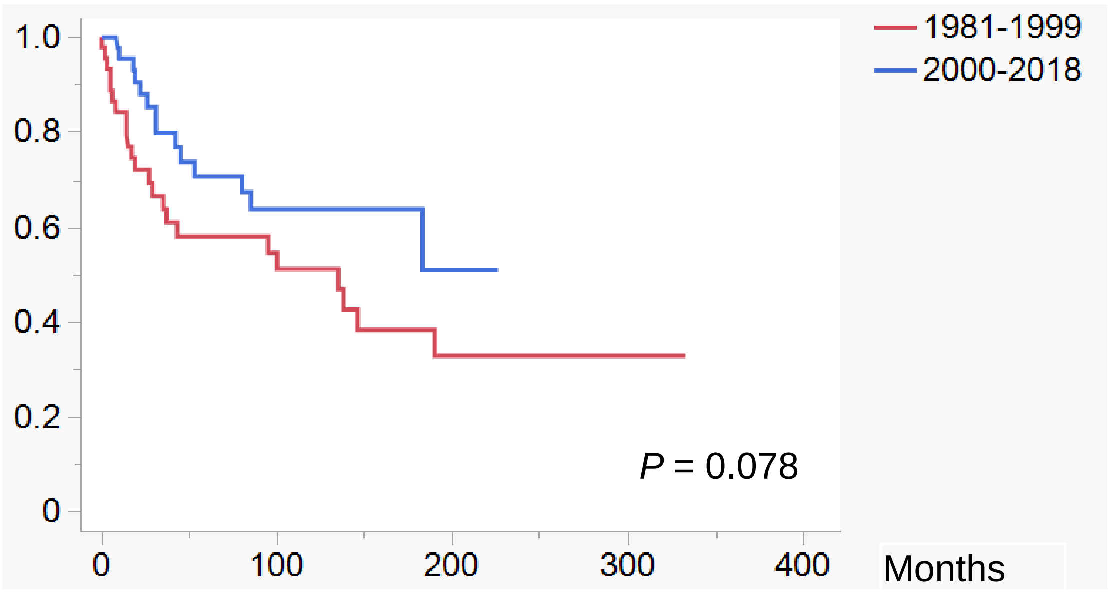

No. at risk:

1981–1999	47	16	5	1	0
2000–2018	48	15	1	0	0

(H) CSS according to the era in pStage III (*n* = 95)

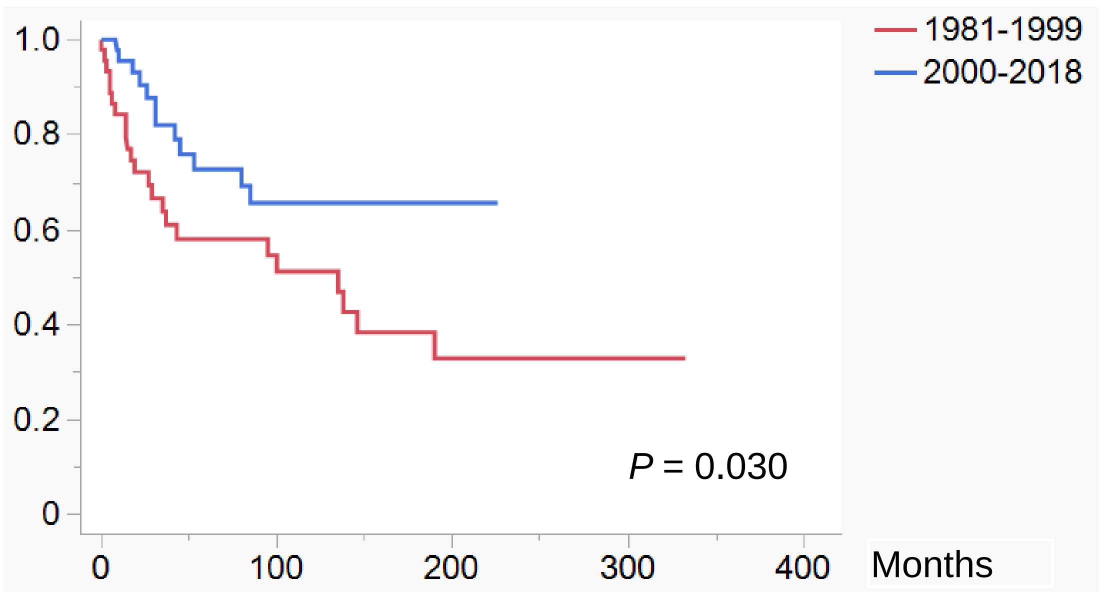

No. at risk:

1981–1999	47	16	5	1	0
2000–2018	48	15	1	0	0

(I) RFS according to the era in pStage III (*n* = 95)

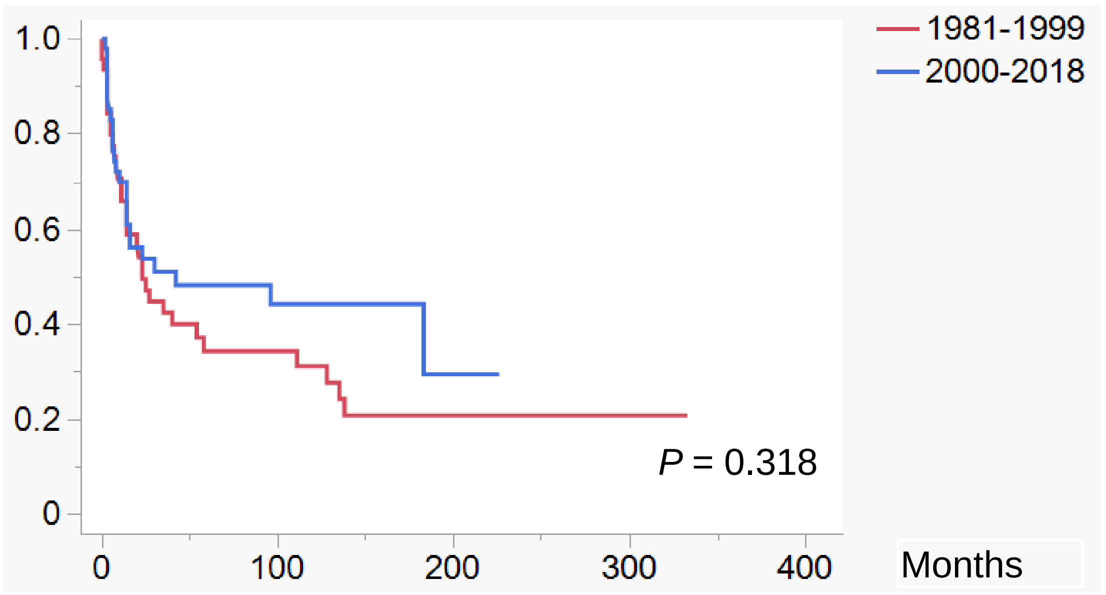

No. at risk:

1981–1999	47	12	4	1	0
2000–2018	48	9	1	0	0

(J) OS according to the era in pStage IV (*n* = 34)

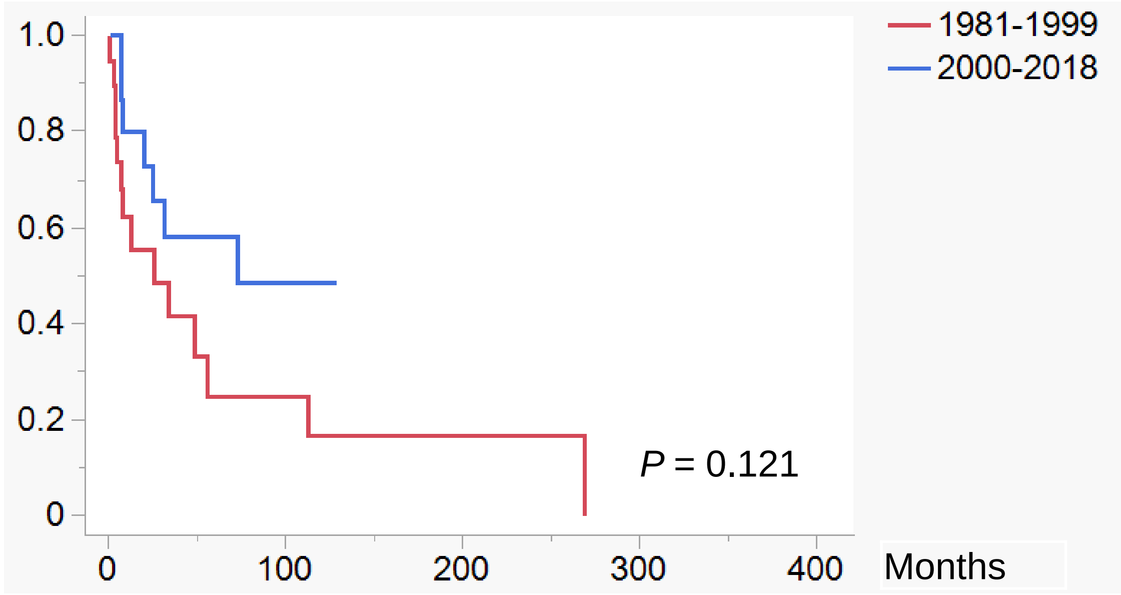

No. at risk:

1981–1999	19	3	2	0	0
2000–2018	15	2	0	0	0

(K) CSS according to the era in pStage IV (*n* = 34)

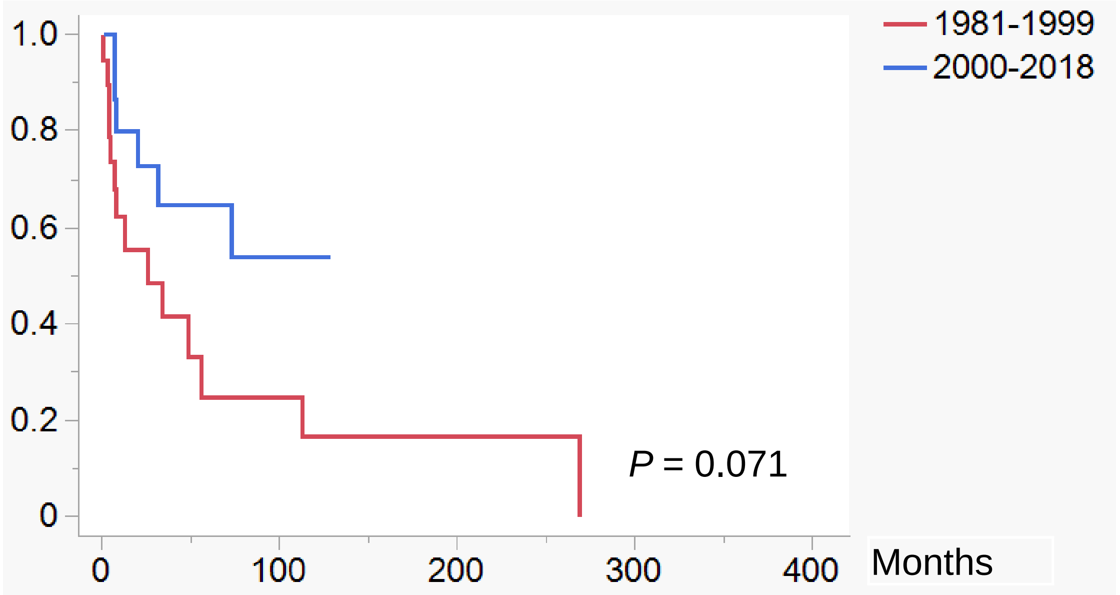

No. at risk:

1981–1999	19	3	2	0	0
2000–2018	15	2	0	0	0

(L) RFS according to the era in pStage IV (*n* = 34)

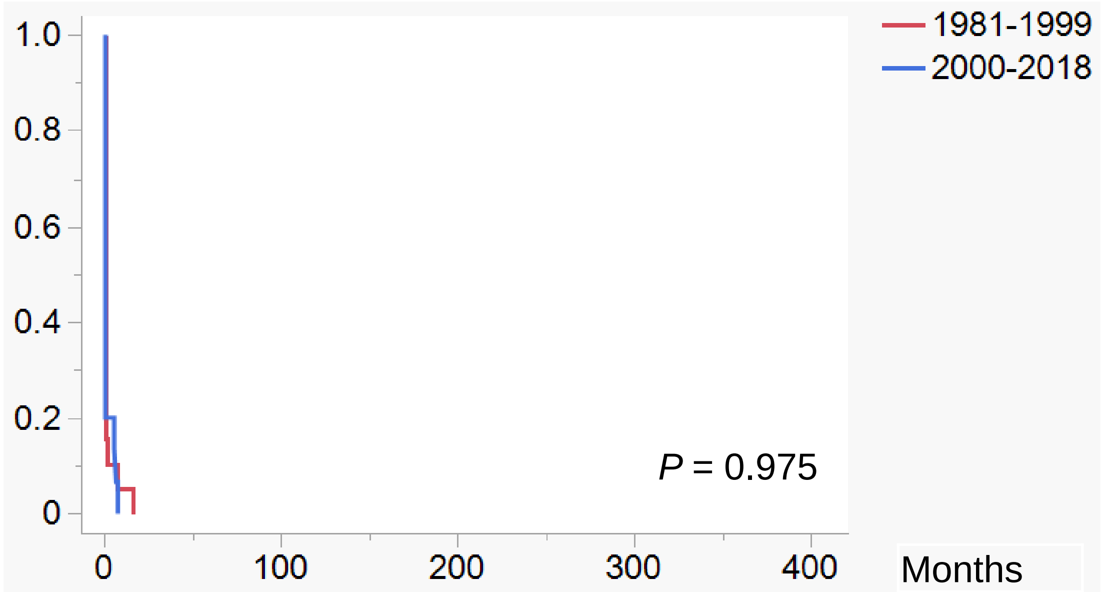

No. at risk:

1981–1999	19	0	0	0	0
2000–2018	15	0	0	0	0
