# Supplementary material for: Survival improvement over time in renal cell carcinoma treated with nephrectomy: A longitudinal propensity score‐matched study
Source: Int J Urol. 2024 Oct 28;32(2):145–50. doi: 10.1111/iju.15610 (PMC11803181; doi:10.1111/iju.15610)
Supplement: Supplementary file 5 — Table S1. [file IJU-32-145-s004.docx]

**Supplementary Table 1.** Univariable and multivariable Cox proportional hazard regression analyses of (A) OS, (B) CSS, and (C) RFS before PSM (*n* = 960).

(A) Cox proportional hazard regression analyses of OS before PSM (*n* = 960).

| Parameter | Cutoff | Univariable | | Multivariable | |
| --- | --- | --- | --- | --- | --- |
|  |  | HR (95% CI) | *P* | HR (95% CI) | *P* |
| Age | ≥ 61 years | 2.15 (1.60 to 2.88) | < 0.001^*^ | 2.30 (1.69 to 3.12) | < 0.001^*^ |
|  | ≤ 60 years | Reference |  | Reference |  |
| Sex | Male | 1.59 (1.10 to 2.29) | 0.014^*^ | 1.39 (0.96 to 2.02) | 0.084 |
|  | Female | Reference |  | Reference |  |
| Era | 1981–1999 | 2.05 (1.53 to 2.75) | < 0.001^*^ | 1.40 (1.00 to 1.96) | 0.049^*^ |
|  | 2000–2018 | Reference |  | Reference |  |
| Surgical procedure | Open | 3.06 (2.00 to 4.67) | < 0.001^*^ | 2.13 (1.34 to 3.39) | 0.001^*^ |
|  | Laparoscopic/Robotic | Reference |  | Reference |  |
| Nephrectomy type | Radical | 3.08 (2.07 to 4.57) | < 0.001^*^ | 1.80 (1.16 to 2.78) | 0.008^*^ |
|  | Partial | Reference |  | Reference |  |
| pStage | III–IV | 6.04 (4.55 to 8.02) | < 0.001^*^ | 4.12 (3.03 to 5.61) | < 0.001^*^ |
|  | I–II | Reference |  | Reference |  |
| Histological type | Non-clear cell | 1.39 (0.94 to 2.05) | 0.102 | 1.69 (1.14 to 2.51) | 0.009^*^ |
|  | Clear cell | Reference |  | Reference |  |

CI, confidence interval; HR, hazard ratio; OS, overall survival; PSM, propensity score matching

^*^ Statistically significant

(B) Cox proportional hazard regression analyses of CSS before PSM (*n* = 960).

| Parameter | Cutoff | Univariable | | Multivariable | |
| --- | --- | --- | --- | --- | --- |
|  |  | HR (95% CI) | *P* | HR (95% CI) | *P* |
| Age | ≥ 61 years | 1.90 (1.39 to 2.60) | < 0.001^*^ | 2.10 (1.52 to 2.91) | < 0.001^*^ |
|  | ≤ 60 years | Reference |  | Reference |  |
| Sex | Male | 1.55 (1.05 to 2.29) | 0.028^*^ | 1.29 (0.86 to 1.92) | 0.213 |
|  | Female | Reference |  | Reference |  |
| Era | 1981–1999 | 2.66 (1.94 to 3.64) | < 0.001^*^ | 1.66 (1.16 to 2.38) | 0.005^*^ |
|  | 2000–2018 | Reference |  | Reference |  |
| Surgical procedure | Open | 4.32 (2.57 to 7.26) | < 0.001^*^ | 2.63 (1.50 to 4.61) | < 0.001^*^ |
|  | Laparoscopic/Robotic | Reference |  | Reference |  |
| Nephrectomy type | Radical | 3.80 (2.40 to 6.01) | < 0.001^*^ | 1.96 (1.19 to 3.23) | 0.008^*^ |
|  | Partial | Reference |  | Reference |  |
| pStage | III–IV | 7.22 (5.32 to 9.81) | < 0.001^*^ | 4.73 (3.40 to 6.59) | < 0.001^*^ |
|  | I–II | Reference |  | Reference |  |
| Histological type | Non-clear cell | 1.38 (0.91 to 2.10) | 0.131 | 1.69 (1.11 to 2.58) | 0.015 |
|  | Clear cell | Reference |  | Reference |  |

CI, confidence interval; CSS, cancer-specific survival; HR, hazard ratio; PSM, propensity score matching

^*^ Statistically significant

(C) Cox proportional hazard regression analyses of RFS before PSM (*n* = 960).

| Parameter | Cutoff | Univariable | | Multivariable | |
| --- | --- | --- | --- | --- | --- |
|  |  | HR (95% CI) | *P* | HR (95% CI) | *P* |
| Age | ≥ 61 years | 1.68 (1.33 to 2.11) | < 0.001^*^ | 1.55 (1.22 to 1.97) | < 0.001^*^ |
|  | ≤ 60 years | Reference |  | Reference |  |
| Sex | Male | 1.55 (1.16 to 2.06) | 0.003^*^ | 1.39 (1.04 to 1.85) | 0.028^*^ |
|  | Female | Reference |  | Reference |  |
| Era | 1981–1999 | 1.63 (1.29 to 2.06) | < 0.001^*^ | 0.99 (0.76 to 1.30) | 0.962 |
|  | 2000–2018 | Reference |  | Reference |  |
| Surgical procedure | Open | 2.28 (1.70 to 3.07) | < 0.001^*^ | 1.74 (1.25 to 2.41) | < 0.001^*^ |
|  | Laparoscopic/Robotic | Reference |  | Reference |  |
| Nephrectomy type | Radical | 2.49 (1.87 to 3.32) | < 0.001^*^ | 1.61 (1.17 to 2.21) | 0.003^*^ |
|  | Partial | Reference |  | Reference |  |
| pStage | III–IV | 7.02 (5.58 to 8.83) | < 0.001^*^ | 5.12 (3.98 to 6.60) | < 0.001^*^ |
|  | I–II | Reference |  | Reference |  |
| Histological type | Non-clear cell | 1.18 (0.86 to 1.64) | 0.306 | 1.35 (0.97 to 1.87) | 0.075 |
|  | Clear cell | Reference |  | Reference |  |

CI, confidence interval; HR, hazard ratio; PSM, propensity score matching; RFS, recurrence-free survival

^*^ Statistically significant
