# Supplementary material for: Survival improvement over time in renal cell carcinoma treated with nephrectomy: A longitudinal propensity score‐matched study
Source: Int J Urol. 2024 Oct 28;32(2):145–50. doi: 10.1111/iju.15610 (PMC11803181; doi:10.1111/iju.15610)
Supplement: Supplementary file 6 — Table S2. [file IJU-32-145-s002.docx]

**Supplementary Table 2.** Univariable Cox proportional hazard regression analyses of OS, CSS, and RFS after PSM (*n* = 466).

| Parameter | Cutoff | OS | | CSS | | RFS | |
| --- | --- | --- | --- | --- | --- | --- | --- |
|  |  | HR (95% CI) | *P* | HR (95% CI) | *P* | HR (95% CI) | *P* |
| Age | ≥ 61 years | 2.14 (1.47 to 3.11) | < 0.001^*^ | 1.98 (1.34 to 2.92) | < 0.001^*^ | 1.78 (1.32 to 2.40) | < 0.001^*^ |
|  | ≤ 60 years | Reference |  | Reference |  | Reference |  |
| Sex | Male | 1.44 (0.87 to 2.38) | 0.160 | 1.51 (0.89 to 2.58) | 0.128 | 1.26 (0.86 to 1.85) | 0.237 |
|  | Female | Reference |  | Reference |  | Reference |  |
| Era | 1981–1999 | 2.40 (1.55 to 3.72) | < 0.001^*^ | 3.01 (1.86 to 4.86) | < 0.001^*^ | 1.68 (1.22 to 2.31) | 0.002^*^ |
|  | 2000–2018 | Reference |  | Reference |  | Reference |  |
| Surgical procedure | Open | 4.88 (2.14 to 11.1) | < 0.001^*^ | 6.90 (2.53 to 18.81) | < 0.001^*^ | 2.63 (1.61 to 4.30) | < 0.001^*^ |
|  | Laparoscopic/Robotic | Reference |  | Reference |  | Reference |  |
| Nephrectomy type | Radical | 2.22 (1.16 to 4.25) | 0.016^*^ | 2.31 (1.16 to 4.57) | 0.017^*^ | 1.97 (1.21 to 3.21) | 0.007^*^ |
|  | Partial | Reference |  | Reference |  | Reference |  |
| pStage | III–IV | 4.69 (3.23 to 6.81) | < 0.001^*^ | 4.89 (3.32 to 7.22) | < 0.001^*^ | 6.05 (4.46 to 8.22) | < 0.001^*^ |
|  | I–II | Reference |  | Reference |  | Reference |  |
| Histological type | Non-clear cell | 2.19 (1.35 to 3.55) | 0.002^*^ | 2.24 (1.36 to 3.69) | 0.002^*^ | 2.07 (1.38 to 3.12) | < 0.001^*^ |
|  | Clear cell | Reference |  | Reference |  | Reference |  |

CI, confidence interval; CSS, cancer-specific survival; HR, hazard ratio; OS, overall survival; PSM, propensity score matching; RFS, recurrence-free survival

^*^ Statistically significant
